# Identification of mechanistic CKD biomarkers in a rat SNx kidney fibrosis model by transcriptomics and proteomics detectable in biofluids

**DOI:** 10.1038/s41598-025-93894-6

**Published:** 2025-04-02

**Authors:** Karin Barnouin, Elisa Tonoli, Clare Coveney, John Atkinson, Margarida Sancho, Andrew Skelton, David J. Boocock, Linghong Huang, Joseph Shephard, Timothy S. Johnson, Elisabetta A. M. Verderio, Breda Twomey

**Affiliations:** 1 UCB Pharma, Slough, SL1 3WE UK; 2 School of Science and Technology, Centre for Systems Health and Integrated Metabolic Research (SHiMR), Nottingham Trent University, Nottingham, NG11 8NS UK; 3 John Van Geest Cancer Research Centre, Nottingham Trent University, Nottingham, NG11 8NS UK; 4 Department of Biological, Geological, and Environmental Sciences, University of Bologna, BIGEA, 40126 Bologna, Italy; 5 Experimental Renal Medicine, Oncology & Metabolism, University of Sheffield, Sheffield, S10 2RZ UK; 6 Present Address: MSD, London, EC2M 6UR UK; 7 Present Address: Gilead Sciences, Oxford, OX4 4GE UK; 8 Present Address: GSK, London, TW8 9GS UK; 9 Present Address: Mestag Therapeutics, Cambridge, CB10 1XL UK

**Keywords:** Diagnostic markers, Systems analysis

## Abstract

The rat sub-total nephrectomy (SNx) is a functional model of general chronic kidney disease (CKD) where the main pathological driver is glomerular hypertension representative of several subtypes of CKD. Comprehensive transcriptomics and proteomics analyses on the SNx rats were performed to identify biomarkers in plasma or urine that correlate with kidney disease and functional kidney loss. Kidneys were subjected to collagen I and III staining for fibrosis scoring, SWATH-MS proteomics and bulk RNA-sequencing transcriptomics, with SWATH-MS also performed on plasma and urine. Differential expression analysis demonstrated significant dysregulation of genes and proteins involved in fibrosis, metabolism, and immune response in the SNx rats compared to controls. Gene ontology analysis of the intersecting genes and proteins from both studies demonstrated common biology between animal cohorts that reached the predefined kidney disease thresholds (serum creatinine > two-fold or proteinuria > three-fold increase over sham-operated). Thirteen significantly differential molecules were detected with consistent directional changes in both omics datasets. These molecules were detected independently in kidney (both RNA and protein) and urine (protein only), but not in plasma. Bioinformatics analysis enabled the identification of mechanistic CKD biomarkers including lumican and collagen alpha-1(III) chain, whose co-expression has previously been both implicated in fibrosis and detected in urine in CKD patients.

## Introduction

Chronic kidney disease (CKD) affects up to 16% of the population across the world and it is associated with significant mortality and morbidity^1^. CKD is strongly linked to diabetes and hypertension. However, environmental factors and genetic background also concur with the aetiology of this complex condition. A common feature of failing kidneys is glomerulosclerosis and tubulointerstitial fibrosis that ultimately causes progressive kidney failure. Fibrotic remodelling is characterised by excess fibrous connective tissue and proliferation of fibroblasts, which is believed to be aberrant wound healing in response to chronic insult from the primary disease. During fibrotic remodelling, fibroblasts not only proliferate but assume an α-SMA positive myofibroblastic phenotype, epithelial cells can either die or undergo epithelial mesenchymal transformation leading to a more stromal extracellular matrix (ECM) producing phenotype while pericytes proliferate and take on myofibroblast characteristics^2^. Overexpression of ECM components, accompanied by metabolic re-programming is a histopathological hallmark of tissue fibrosis. The deposition, accumulation, increased crosslinking and stabilisation of collagens and fibronectin result in the hardening and scarring of tissues. These changes cause pathological remodelling of the organ which lead to its failure^3,4^. The development of tubulointerstitial fibrosis is the best predictor of progression^5^.

Initial detection of CKD is typically by use of protein dipstick in the urine, with subsequent characterisation and tracking of the stage of disease (CKD stage 1–5) determined by assessment of kidney function. This normally uses serum creatinine levels to estimate the glomerular filtration rate (eGFR) using the Modification of Diet in Renal Disease (MDRD) or CKD Epidemiology Collaboration (EPI) equations. More detailed assessment of stage also incorporates albuminuria measurements with high albuminuria being indicative of more rapidly progressing disease^6,7^.

Kidney biopsy remains the gold standard to diagnose fibrosis within the kidney; however, it not only causes risk and discomfort to the patient but is an imperfect test, susceptible of sample bias and only useful when fibrosis is fully established. Moreover, the biopsy approach neither provides an early diagnosis nor a prognosis response. Importantly, it also limits the development of new anti-fibrotics due to the long time required to determine the effectiveness of a therapeutic fibrosis development.

Serum creatinine and proteinuria levels constitute the main biofluid biomarkers for detection and monitoring of CKD. However, these do not reflect all aspects of kidney disease pathophysiology (for example, kidney tubular health), do not always predict GFR decline, and are late disease markers requiring significant damage to occur. Several recent studies have tried to identify earlier, better, more predictive, and reliable markers of kidney disease in serum and urine to enable both clinical management and clinical trials^8–12^. The identification of progression or mechanistic biomarkers and the underlying pathobiological processes in experimental animal models could enable their use in drug discovery and the translation of pre-clinical to clinical research.

The application of experimental models of CKD are useful for pre-clinical evaluation. However, because CKD progresses in humans over many years with a complex mix of genetics, environmental, age, sex, and lifestyle elements as underlying risk factors, it has been extremely challenging to mimic disease progression with animal models, that at the same time allow a fast and reliable assessment of disease progression or stability. Many experimental models have been developed that can be performed within acceptable time frames, have functional measurements relatable to human kidney disease, and that induce morphological and biochemical changes at the organ level translatable to the clinic, but all have some limitations. The models that enable readout of functional parameters while also displaying the collagen ECM accumulation in kidney tissue are most relevant for clinical translation.

Several kidney damage models such as the mouse unilateral ureteral obstruction (UUO), mouse and rat Adriamycin, mouse aristolochic acid nephropathy (AAN) and rat subtotal nephrectomy (SNx) models have been employed by us and others to simulate CKD^13–20^. The SNx model is generally accepted as a general model that mimics many aspects of human CKD such as focal segmental glomerular sclerosis (FSGS), diabetic nephropathy (DN), membranous nephropathy (MN), IgA nephropathy (IgAN), membranoproliferative glomerulonephritis (MPGN), polycystic kidney disease (PKD), membranous glomerulonephritis (MGN) and rapidly progressive glomerular nephritis (RPGN). It displays some of the histological and pathobiological features typical of changes of focal segmental glomerular sclerosis^21^ with an increase in proteinuria and loss of kidney function associated with tubulointerstitial involvement. CKD in this model is driven by a combination of glomerular hypertension and inflammation, that are key factors in human disease progression. SNx rats also have cardiac defects, one of the co-morbidities for human CKD^20,22^. The model therefore provides a platform with ready access to biofluids and tissues enabling the identification of disease relevant biomarkers.

In this investigation we have therefore aimed to use the SNx model to identify mechanistic markers of CKD development (fibrosis) that are detectable in biofluids and associated with changes in tissue biology, rather than those of plasma that appear in biofluids resulting from fibrotic renal structure deterioration such as currently used proteinuria and serum creatinine. It is anticipated that these could reflect the active nature of disease in humans and be employed as early diagnostic or progression biomarkers.

We have performed two independent rat SNx experiments and tracked disease progression by proteinuria and serum creatinine. Plasma, urine, and kidney tissue were analysed at predefined disease criteria, by label-free quantitative proteomics using the data-independent (DIA) analysis technique called sequential window acquisition of all theoretical fragmentation spectra (SWATH) mass spectrometry (MS) (SWATH-MS). This type of MS acquisition method provides greater depth in protein identification and better quantification than data-dependent analysis (DDA) analysis, especially when the data is interrogated by an in silico generated library^23,24^. Kidney tissues were concurrently analysed by bulk RNA-seq transcriptomics. The readouts of both omics’ experiments were compared and integrated, to identify strong reproducible disease biomarkers symptomatic of disease development in the rat SNx model of CKD.

## Results

### Preparation of diseased rat kidneys for omics analyses

**Fig. 1:**
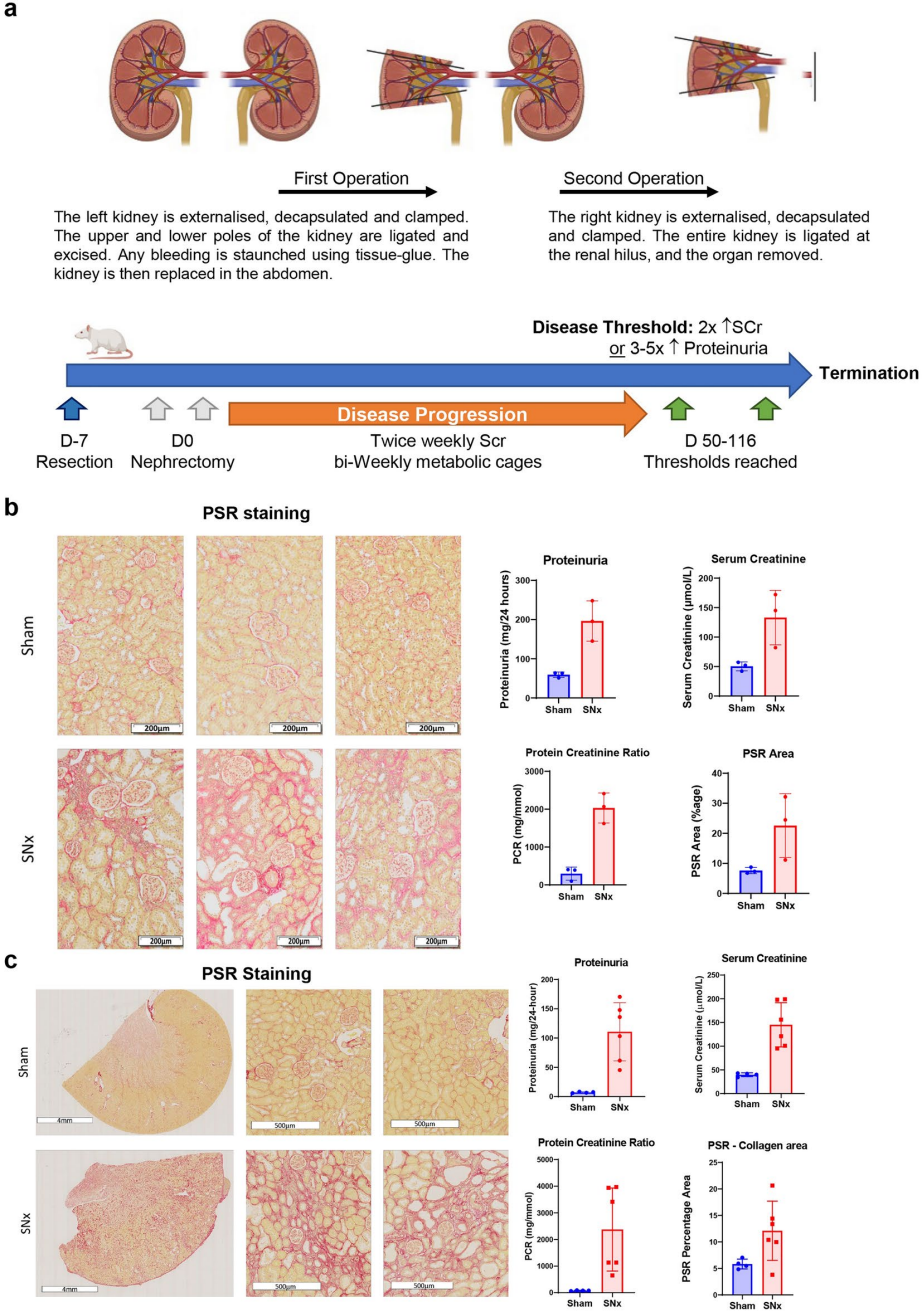
Characterisation of rat SNx study samples. (**a**) The surgical procedure in rats termed sub-total nephrectomy (SNx) where 5/6 of the functioning kidneys are removed and post-surgery the animals develop failing kidney function from day 50 onwards, depicted on a timeline with monitoring of the failing kidneys using a combination of serum creatinine and proteinuria measurements. (**b**) Study 1 kidney sections from either sham (n = 3) or SNx rats (n = 3), stained with Picrosirius Red (PSR) and the PSR-stained kidneys were scanned, the percentage of PSR area plotted alongside the other functional kidney readouts, Proteinuria, Serum creatinine and Protein creatinine ratio. Scale bar: 200 µm. (**c**) Study 2 kidney sections from 1/4 sham rats or 2/6 SNx rats, stained with PSR and PSR-stained kidneys from all rats were scanned, percentage of PSR area plotted alongside the other functional kidney readouts, Proteinuria, Serum creatinine and Protein creatinine ratio. Scale bar: 4 mm or 500 µm, as indicated.

As the objective of this work was identification of kidney disease biomarkers representative of progressive phenotypes that could correlate with biomarkers in the more accessible serum and urinary compartments, we thought it imperative to synchronise the animals for disease development, with tissue recovery occurring when disease thresholds had been reached. Two separate SNx studies (named study 1 and study 2) were performed at two independent sites with similar pre-defined disease monitoring criteria as summarised in Fig. 1a. Animals were only terminated when they reached the disease threshold functional levels of twofold increase in serum creatinine or threefold increases in proteinuria over sham-operated controls. However, the time to reach these disease thresholds varied between the studies where study 1 animals were terminated between days 81–116 and study 2 animals between days 52–98, reflecting variability not only between groups but also between individual animals within the same study. All the animals in study 1 were included in the analysis. However, only six out of eighteen rats reached the predetermined threshold in study 2. Representative kidney sections, stained for collagens I and III content using pico-sirius red (PSR) from both study 1 and 2, are shown in Figs. 1b, c, respectively. Urine and serum samples from both studies collected before termination were analysed for serum creatinine, proteinuria, and urinary proteinuria creatinine ratio as measurements of kidney function and glomerular damage (Figs. 1b, c), as well as processed for proteomics. Kidney sample preparation for transcriptomic and proteomic analyses were similar between the two studies.

### RNA-Seq transcriptomics–global observations

**Fig. 2:**
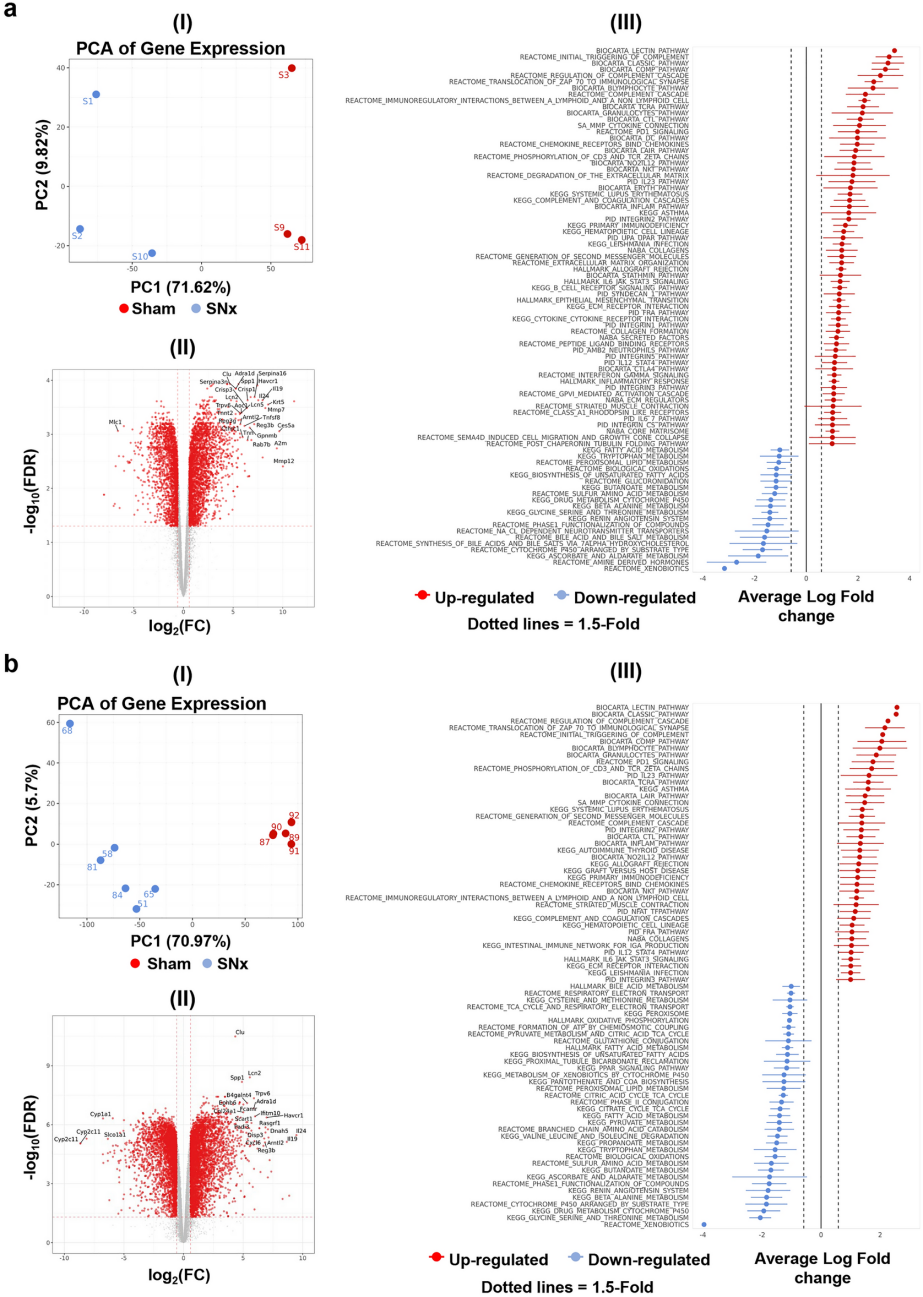
RNA seq analysis of kidneys from SNx compared to sham rats. PCA (I), differential gene analysis Volcano plot (II) and Gene Pathway Ontology CAMERA Functional enrichment analysis (III) were performed for both study 1 (**a**) and study 2 (**b**).

Bulk-RNA seq were performed on rat SNx, Adriamycin, and UUO kidney tissues. About 12,000 genes were profiled in each RNA-sequencing experiment (Table 1, Supplementary Fig. 1, and Supplementary data 1). Unsupervised PCA analysis of the two SNx studies transcriptomics data showed that, despite intra- and inter-study variability, the diseased SNx animals could be separated from sham (healthy) controls (Fig. 2a-I, b-I). In study 1, over 4000 proteins changed significantly (FDR < 0.05) after SNx treatment with over 1700 genes down- and up-regulated (Fig. 2a-II, Table 1). In study 2, over 4700 genes were significantly modulated (FDR < 0.05) with over 2800 genes that were down- and up-regulated (Fig. 2b-II, Table 1). Gene Pathway Ontology analysis showed that the most abundantly upregulated genes are represented by gene products involved in complement, immunological, and STAT signalling, associated with response to inflammation and ECM organisation pathways. Most of the significantly down-regulated genes are involved in the metabolism of amino acids, fatty acids or in mitochondrial processes (Fig. 2a-III, b-III).

**Table 1:** Number of total, unchanged and significantly changing FDR < 0.05 proteins in SWATH-DIA and genes in RNA-seq experiments.

Differential expression	Proteins				Transcripts	
	Kidney study 1	Kidney study 2	Plasma study 2	Urine study 2	Kidney study 1	Kidney study 2
Total	6329	6871	488	632	12,714	11,533
Unchanged	5151	2593	444	184	8618	5667
Downregulated	220	659	2	207	1797	2812
Upregulated	958	3619	42	241	2299	3054

Heatmap comparison of transcriptomics profiles of rat SNx with Adriamycin and UUO kidney disease rat models revealed that although most of the genes that are up- or down-regulated in each experiment are similar, the magnitude of change differs between them. Overall, genes involved in T-cell inflammatory processes are upregulated in both studies, whereas down-regulated genes are involved in metabolism. Expectedly, ECM modelling pathways implicated in kidney fibrosis regulation are more upregulated in the aggressive UUO and Adriamycin models than in both SNx studies (Supplementary Fig. 1).

## DIA-SWATH proteomics – global observations

**Fig. 3:**
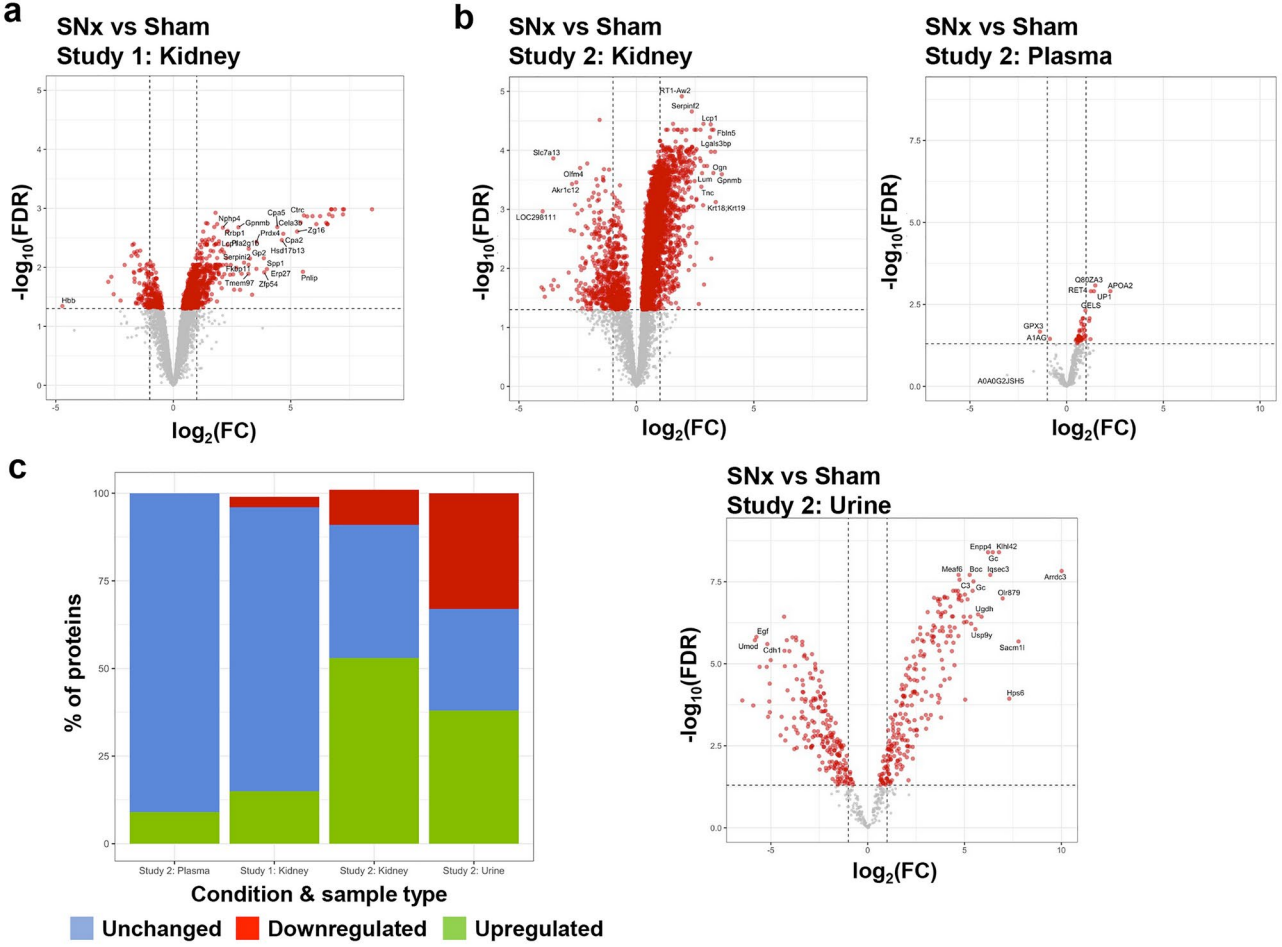
Significantly changed proteins in SNx compared to sham rat kidneys. Volcano plots showing up- and down-regulated proteins in study 1 kidney (**a**) and study 2 kidney, plasma and urine (**b**). (**c**) Summary of protein counts expressed as percentages of downregulated (red), upregulated (green) and unchanged (blue) proteins compared to total number of proteins quantified in each sample.

Over 6000 proteins were identified in the rat kidneys, and more than 450 proteins in study 2 plasma and urine were identified (Table 1, Fig. 3, Supplementary Fig. 2, and Supplementary data 1). As with the transcriptomics data, PCA analysis of the protein expression could clearly separate Sham (Healthy) and SNx rats for both kidney and urine datasets. In contrast, the separation was less distinct for plasma (Supplementary Fig. 3).

## Gene ontology analysis of proteins

To gain a deeper understanding of the changes in the biological processes and molecular functions as well as in which cellular compartment these occur in CKD, Gene Ontology (GO) analysis was performed on the up- and down-regulated genes or proteins that were identified in both the proteomic and transcriptomic data. The web-based application Rivigo, that employs an algorithm that clusters GO terms according to semantic similarity, was used to summarise the data. The data was visualised as TreeMaps where the larger the square, the larger the log10 p-value, the more significant the enrichment. For the up-regulated kidney proteins, post-SNx processes involved in stress and structural pathways were the most significantly enriched, for example, regulation of collagen fibril organization and cellular response to reactive nitrogen species (Supplementary Fig. 6a and data 2). The most enriched GO biological processes for down-regulated kidney proteins post SNx included responses to translation, oxidative stress, detoxification, cell matrix adhesion and metabolic pathways (e.g., glucose 6-phosphate metabolic process) (Supplementary Fig. 6b and data 2). In urine the identified down-regulated proteins have been shown to play roles in immune response, proteolysis, transport, or cell adhesion. Up-regulated proteins in urine are involved in endopeptidase activity, small molecule transport, plasminogen activation or cell structure while in plasma up-regulated proteins are implicated in complement activation, lipid metabolism, transport, and cytolysis (Supplementary Fig. 4 and data 2).

**Fig. 4:**
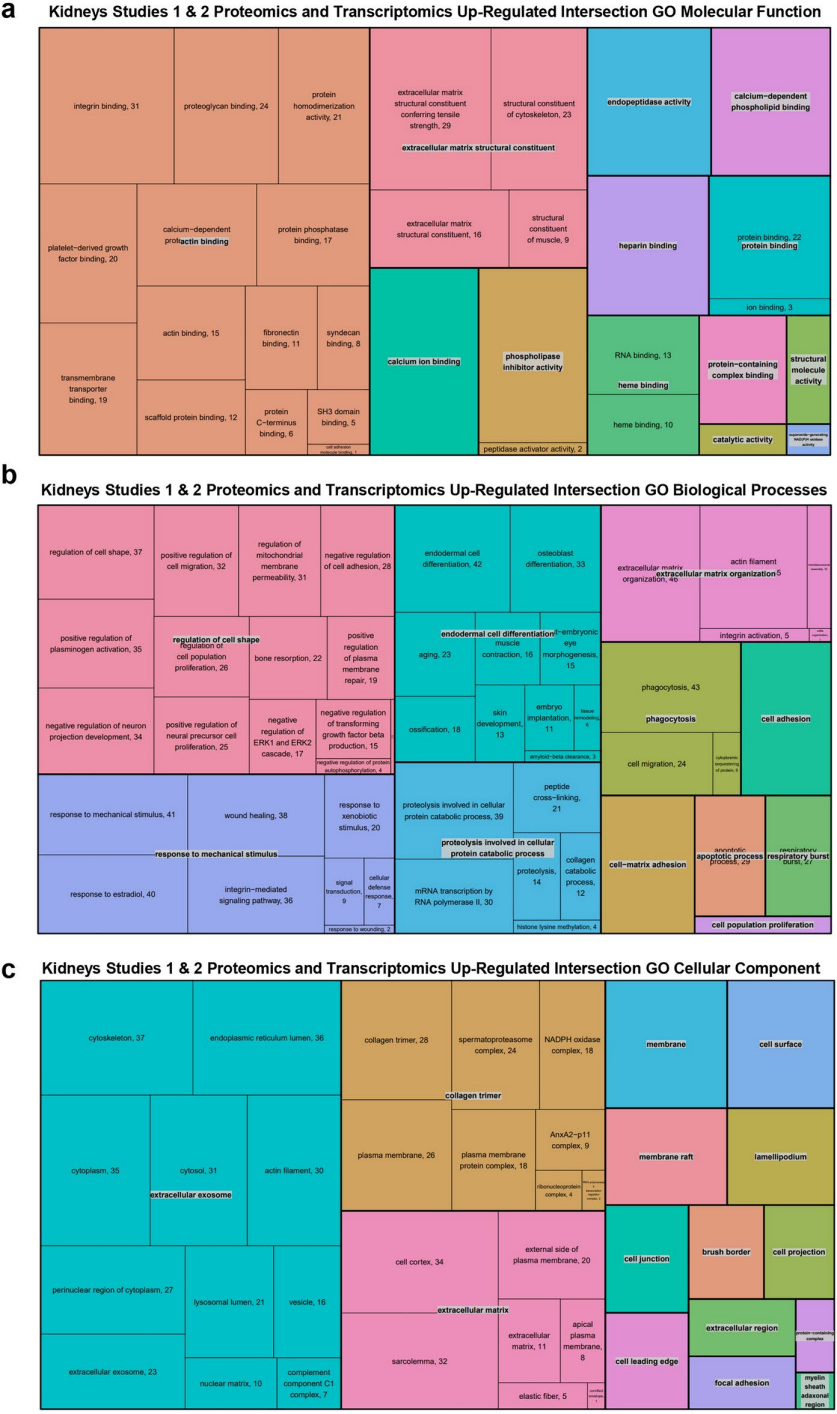
GO of up-regulated intersecting proteins and genes in kidney. TreeMaps showing enriched Molecular Functions (**a**), Biological processes (**b**) and Cellular compartments (**c**) of the significantly up-regulated proteins/genes in the kidneys of both studies.

We then performed GO analysis of the intersecting proteins. The most significantly enriched biological processes in the up-regulated proteins in proteomics kidney studies were those involved in catabolic, metabolic, cell polarity and immune responses. Notably, the most CKD relevant significantly enriched GO biological processes were the proteins and genes that were in common between study 1 and 2 in the kidney proteomics and transcriptomics. Those proteins are involved in cell adhesion, cell differentiation, matrix organisation, cell proliferation, phagocytosis, and immune system processes. Furthermore, the cellular compartments were similar in study 1 and 2 kidneys in the proteomics and transcriptomics data and mainly corresponded to the localisation of proteins that play a role in cell shape and structure. Please refer to TreeMaps and tables for more details (Fig. 4, Supplementary Fig. 5 and 6, and data 2).

### Translatable CKD biomarker identification by data integration

**Fig. 5:**
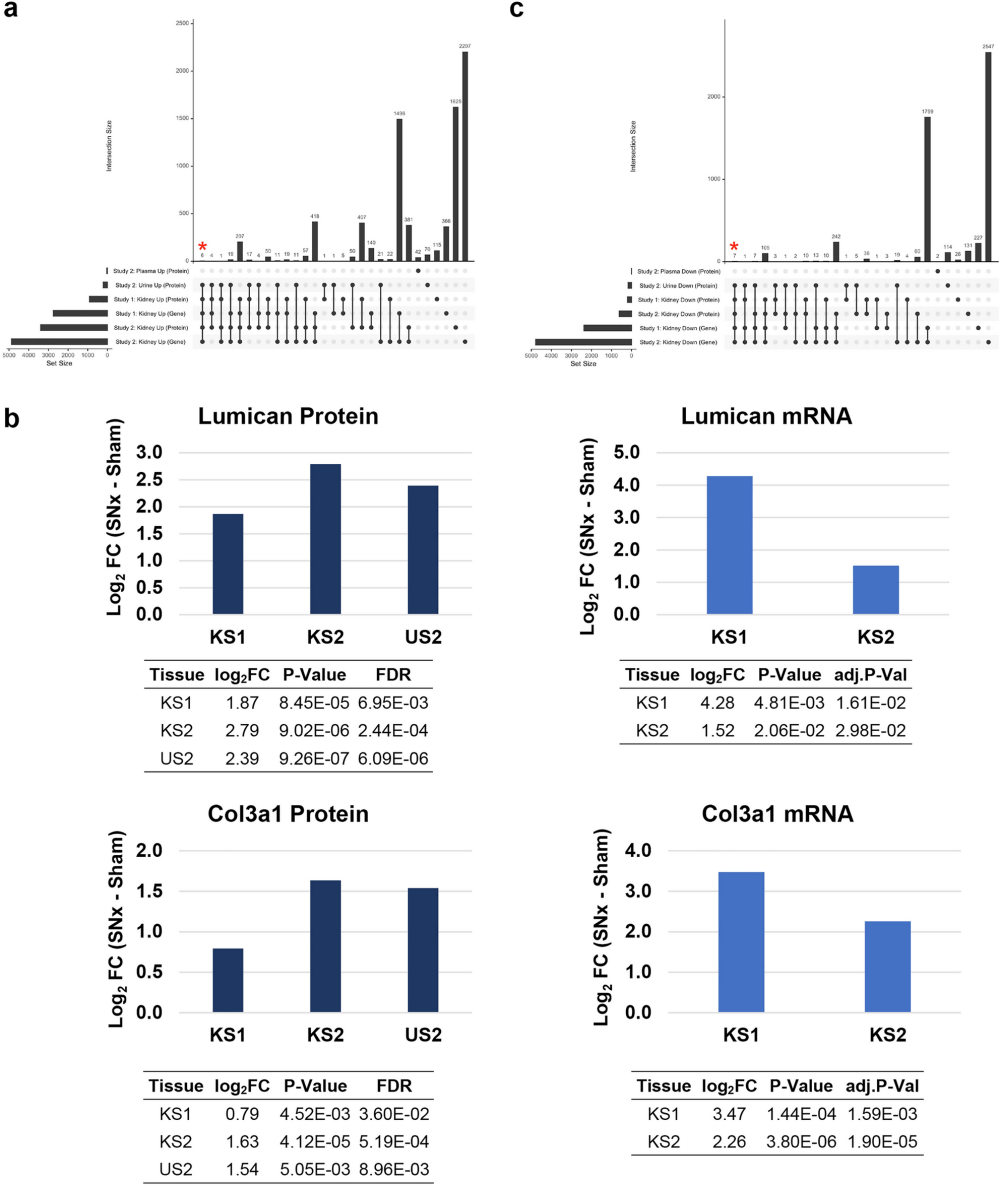
Proteomics and gene expression intersections. (**a**) Upset plots showing up-regulated proteins/genes in common between study 1 and study 2 proteomics-transcriptomics data (kidneys, plasma, urine; FDR < 0.05). Red asterisk indicates proteins/genes up-regulated in all analyses apart from plasma. (**b**) Protein and gene expression of lumican and collagen type III alpha1 chain (Col3a1). (**c**) Upset plots showing down-regulated proteins/genes in common between study 1 and study 2 proteomics-transcriptomics data (kidneys, plasma, urine; FDR < 0.05). Red asterisk indicates proteins/genes down-regulated in all analyses apart from plasma.

Upset plots were used to identify the intersecting proteins and genes between the up- or down-regulated genes or proteins in both proteomics and transcriptomics methods (Fig. 5, Supplementary Fig. 5). Thirteen proteins were significantly differentially regulated (six upregulated, seven downregulated) at both protein and transcript levels in kidney and urine in all the analyses. The upregulated proteins were lumican (Lum), profilin 1 (Pfn1), collagen alpha-1(III) chain (Col3a1), complement component C6 (C6), collagen type VIII alpha1 chain (Col8a1) and Immunoglobulin Heavy Constant Mu (Ighm) (Fig. 5a, b, Supplementary Fig. 7a). The downregulated proteins were solute carriers Slc7a13 and Slc3a1, gamma-glutamyl transferase (Ggt1), the hydrolases alpha/beta hydrolase domain containing 14b (Abhd14b), Erythrocyte Membrane Protein Band 4.1 Like 3 (Epb41l3), trimethyllysine hydroxylase (Tmlhe) and the dipeptidyl peptidase 4 (Dpp4) (Fig. 5c, Supplementary Fig. 7b). Although these proteins are commonly detected in plasma, none of them passed the FDR 0.05 threshold for significant fold change in this biofluid. All these proteins except for DPP4 were also found to be similarly differentially regulated on the transcript level in the kidneys of Adriamycin and UOO rat models (Supplementary transcriptomic and proteomics Limma analysis data).

## Discussion

CKD animal models are of value in the preclinical setting for identifying biomarkers and surrogate target engagement biomarkers^25^. Several models have been developed but inherently display differences in their capacity to monitor functional kidney data alongside quantitative gene and protein changes. Selecting the right model of CKD is crucial in pre-clinical research to test interventions aimed at controlling CKD. Transcriptomic comparison of different models performed here has shown variation in the quantitative and qualitative gene expression profiles of TGF beta and SMAD pathways known to be modulated in fibrosis and extracellular matrix remodelling (Supplementary Fig. 1). Although none of the models are perfect predictors of human disease, the choice of model and rodent depends on the specific project and therapeutic target requirements, such as the option for functional correlation, representation of distinct pathway changes, and model duration, which can distinguish acute versus chronic phases of disease progression. The ideal model for biomarker discovery would enable monitoring of the molecular changes non-invasively as the disease evolves.

The SNx model was selected because it is the best characterised and has the capacity for real-time functional monitoring of disease progression using serum creatinine and proteinuria measurements before termination. Typically, widespread glomerulosclerosis and tubulointerstitial fibrosis gradually develop, mimicking the progressive renal failure in humans consequent to loss of functional renal mass. However, the accurate interpretation of the data generated from the models is not straightforward because of the high variability of the response of the rats to the subtotal nephrectomy surgery. The analysis of the two SNx models independently generated in this study demonstrated both inter- and intra-model differences in the time it took to reach a specific disease threshold determined by functional analyses. The variability between animals was evident in the Principal Component Analysis (PCA) plots of both the RNA-seq and proteomics, where the healthy rats cluster close together, whereas the SNx rats are in comparison further apart (Fig. 2a, b and Supplementary Fig. 3). Integrating the omics data acquired from the two independent SNx studies has enabled the identification of CKD and fibrosis-specific biomarkers. Many genes were differentially expressed at both protein and transcript levels in kidney tissue in the two SNx studies. Many proteins also changed significantly in the urine, but not in plasma. Among those changing, six proteins were upregulated at both the protein and transcript level in the kidney and urine in all the analyses, including in the RNAseq studies performed in UUO and Adriamycin kidney tissues: Lum, Col3a1, Col8a1, Pfn1, C6, and Ighm. Interestingly, Lum, a proteoglycan, and Col3a1, co-express and regulate keratan, TGFbeta2, and FasL that in turn, modulate MDM2-p53, apoptosis, as well as TGF-beta signalling pathway^26^. These pathways have previously been implicated in growth suppression and cell adhesion (KEGG pathway proteoglycans in cancer), lung disease^27^, kidney (Supplementary Fig. 8), and other fibrotic diseases^28^

Notably, Lum mRNA levels has been found to be present in significantly higher levels in RPGN, DN, IgAN, FSGS, MGN, lupus nephritis (LN) and hypertensive nephropathy (HT). In the same study, lumican protein stained positive in DN patients^29^**.** Interestingly, lumican was only detected in the urine of stage 3 CKD patients and not in healthy controls in a glycoproteomics study indicating that it could be used as an early biomarker^30^. Furthermore, Lum was recently identified as an early diagnostic biomarker for children with Alport Syndrome^31^. Col3A1 kidney mRNA and protein in urine was also found to be elevated in a puromcyin aminonucleoside (PAN)- induced nephropathy rat model^32^. It is thought that during ECM remodulation fragments of collagen degradation products are secreted in urine preceding proteinurea, and therefore could be used as an early marker of progressive renal fibrosis^33^. Indeed, 55 fragments of Col3A1 were detected in a urine proteomic study of CKD patients^34^. Downregulation of Pfn1 has been shown to be regulated by CK2 and has a protective effect against kidney fibrosis^35^. In contrast, C6, Col8a1, and Ighm are all involved in the complement pathway, and their upregulation is related to a general immune response to injury and disease (Figs. 4b and 7a) and have also previously been shown to contribute to CKD progression^36^.

Proteins and genes downregulated in urine as well as kidneys (but not plasma) at both the protein and transcript levels were Slc7a13, Slc3a1, Ggt1, Abhd14b, Epb41l3, Tmlhe and Dpp4. Slc7a13 and Slc3a1 are both involved in amino acid transport and are highly expressed in kidneys. Downregulation or dysfunction of solute carriers has previously been observed in kidney disease^37^. Ggt1 catalyses the transfer of glutathione to small molecules and generally plays a protective role against toxins and oxidative stress, but also specifically in kidneys^38,39^. Tmlhe is involved in amine, polyamine, and carnitine biosynthesis. Increased levels of L-carnitine have been shown to attenuate kidney fibrosis development^40^. Interestingly, Dpp4 acts as a serine endopeptidase and inhibitors against its activity are used to treat diabetes and has also been shown to metabolise collagen in kidney^41^. Epb41l3 is an adaptor protein that interacts with cytoskeletal proteins and is believed to modulate the activity of methyl transferases such as PRMT3 and PRMT5^42,43^. DNA-methylation is thought to contribute to epigenetic causes of diabetic kidney disease^44^. Taken together, all these CKD biomarkers identified herein have been shown to be modulated during the development of fibrosis.

The aim of our analysis was to identify proteins that were detectable in all the transcriptomics and proteomics datasets that could be used as candidate mechanistic CKD markers. From the CKD biomarker candidates detectable in urine in the proteomics study conducted by Kim et al.^45^ we also found significantly increased levels of Pros1 and Tf, but only in study 2 kidney and urine proteomics. Interestingly, established CKD marker NGAL1 (LCN2) transcript was significantly increased (log_2_ 5-fold change (FC) (or 32 FC) in both studies 1 and 2). Protein levels were up by log_2_ 1.3 FC (or 2.5 FC) and log_2_ 2.3 FC (or 5 FC) in studies 1 and 2, respectively, but not in urine. Whereas KIM1 (Havcr1) was only significantly up in study 2 (log_2_ 2.7 FC or 6.5 FC) kidney proteomics but not in urine proteomics or kidney transcriptomic. These observations indicate that KIM1 and NGAL1 may not be suitable as urine prognostic markers.

In summary, the data presented here is, to our knowledge, the first time that SWATH-MS data has been collected on SNx rat kidney tissue, plasma, and urine and systematically compared. In addition, there are no previous reports that compare the proteome profile with mRNA expression levels in SNx models. Whilst mRNA and protein expression levels do not always correlate, analysis of the differential expression of the proteins and genes in all the datasets and enriched gene ontologies have enabled the identification of Lum and Col3a1 as relevant translatable mechanistic CKD biomarkers that can be detected in urine as a single assay. The possibility of developing a multiplexed assay that tracks changes in several of the identified proteins could offer a platform for detecting early signs of progressive human renal disease before glomerular lesions and tubulointerstitial lesions become measurable. Furthermore, it could provide a signature to rapidly monitor response to anti-fibrotic therapies in clinical studies or the effectiveness of existing therapies earlier in clinical care. Finally, this unique multi-omics profiling data of renal tissue and biofluids post-SNx also serves as a repository together with others^46^ for scientists in the field to obtain information that can be reanalysed and validated using orthogonal or targeted methods.

## Methods

### Subtotal nephrectomy rat, UUO mouse, and adriamycin mouse model generation

Subtotal Nephrectomy (SNx) was performed on male Wistar Han rats (200–400 g Charles River, UK) essentially as described^22,47^ using 2/3 resection of the right kidney followed two weeks later by left kidney nephrectomy. Study 1 (performed at UCB Pharma) contained 3 non-op and 3 SNx rats. Study 2 (performed at the University of Sheffield) had 5 sham and 18 SNx operated rats. Serum creatinine was measured weekly and proteinuria biweekly with animals culled using a Schedule 1 method when proteinuria reached > 3–fivefold increase or serum creatine reached > twofold increase over sham-operated. At termination, 24-h urine and plasma samples were collected and snap frozen at − 80 °C. Following Schedule 1 killing, kidney tissue was recovered, with 1⁄4 fixed in NBF for histology and the three other 1⁄4 snap frozen in liquid nitrogen for SWATH or Transcriptomics.

For comparison to other models, UUO, and Adriamycin mouse models of CKD were generated alongside the SNx at UCB Pharma under project licence (30/3051). Procedures were performed as described in^48,49^, respectively. Detailed methods for the generation of the models and histology analyses can be found in the Supplementary Information 1 methods section.

The studies were carried out under license according to the regulations of housing conditions, husbandry, and procedures laid down by Her Majesty’s Government, UK (Animals Scientific Procedures Act 1986). Study 1 was performed at UCB Biopharma under project license 30/3051 and study 2 at the University of Sheffield under project license (PBE09C70E). This study was reported according to ARRIVA guidelines.

### Kidney clinical measurements

SNx rats were placed in metabolic cages to collect 24-h urine samples. Urine was centrifuged to remove any sediment from food or faeces prior to analysis. Protein content in urine was determined using a modified Lowry method^50^. Urine creatinine was measured using an enzymatic creatinine assay (Crystal Chem, USA, Cat: 80,340) and serum creatinine a StatSensor Xpress (Nova Biomedical, USA) with creatinine Stat Strips. The protein:creatinine ratio was calculated as mg protein per mmol of creatinine.

### Bulk RNA sequencing

Material was quantified using RiboGreen (Invitrogen) on the FLUOstar OPTIMA plate reader (BMG Labtech) and the size profile and integrity analysed on the 2200 or 4200 TapeStation (Agilent, RNA ScreenTape). RIN estimates for all samples were between 1.3 and 7.8. Input material was normalised to 30ng prior to library preparation. The RNA was depleted of ribosomal RNA using NEBNext rRNA Depletion Kit (NEB, Human/Mouse/Rat) following manufacturer’s instructions. Strand specific library preparation was completed using NEBNext Ultra II mRNA kit (NEB) following manufacturer’s instructions. Libraries were amplified (14 cycles) on a Tetrad (Bio-Rad) using in-house unique dual indexing primers (based on^51^). Individual libraries were normalised using Qubit, and the size profile was analysed on the 2200 or 4200 TapeStation. Individual libraries were normalised and pooled together accordingly. The pooled library was diluted to ~ 10 nM for storage. The 10 nM library was denatured and further diluted prior to loading on the sequencer. Paired end sequencing was performed using a NovaSeq6000 platform (Illumina, NovaSeq 6000 S2/S4 reagent kit v1.5300 cycles).

### RNA-seq data analysis

Raw sequencing data was initially assessed with the FastQC and MultiQC utilities. Transcripts were quantified using the Rn6 reference transcriptome, and pre-built selective alignment index from RefGenie. Quantifications were summarised to the length scaled TPM unit via tximport and voom transformed with the Limma framework. Differential expression analysis was performed with Limma, and functional analyses with the CAMERA function and Mitch framework, using MSigDB reference gene sets^52^.

### Proteomics sample preparation and LCMS SWATH analysis

SNx and sham kidney sections were processed as previously described^53^ with minor modifications. Specifically, after kidneys were lysed in lysis buffer [9.5 M urea, 2% (w/v) DTT, 1% (w/v) *N*-octyl-β-glucopyranoside, protease inhibitors; 1 mL of buffer every 100 mg of tissue], protein extracts (75 μg) were vacuum concentrated to dryness and stored at − 20°C for 16 h. Samples were then resuspended in solubilisation buffer [5% SDS (w/v), 100 mM triethylammonium bicarbonate (TEAB), pH 7.55] and proteins digested by S-Trap (PROTIFI, US) according to manufacturer’s instructions. Urine samples were thawed on ice and supplemented with protease inhibitors (Roche), centrifuged at 5000 × g, passed through a 0.45 μm syringe filter, concentrated and desalted using centrifugal concentrator tubes (Vivaspin 500, 5 kDa MWCO, Sigma-Aldrich). Urinary proteins underwent reduction, alkylation, and trypsin digestion for 16 h as previously described^53^. Plasma samples were thawed on ice and supplemented with protease inhibitors (Roche), then cleared using Spin-X® Centrifuge Tube Filters (Corning) and 50 μg processed as described above. The resulting peptides were vacuum dried and stored at -20° C until reconstitution for MS analysis.

6.6 µg of sample was injected and trapped onto a YMC Triart-C18 pre-column (5 × 0.3 mm, 3 µm) at a flow rate of 10 µL/min for 2 min prior to switching the valve to run the gradient elution through a YMC Triart-C18 analytical column (150 × 0.3 mm, 3 µm, 5 µL/min) in line with the Sciex TripleTOF 6600 via a Duospray Source using a 50 µm electrode in positive mode, + 5500 V. The following linear gradients were used for SWATH-MS acquisition, mobile phase B increasing from 3 to 30% over 38 min, 30% to 40% over 5 min, 40% to 80% over 2 min for wash and re-equilibration (total run time 57 min). Data independent (SWATH) acquisition was performed using 100 variable SWATH windows (optimised on sample type) with the following parameters: TOFMS *m/z* 400–1250, MSMS *m/z* 100–1500, 25 ms accumulation time; 2.6 s total cycle time.

### Label-free SWATH mass spectrometry data analysis

SWATH mass spectrometry data was converted to MZML using MSConvert (Proteowizard MS Covert Version: 3.0.21075-41cb77451) and processed using DIA-NN (ver1.8) (Demichev, 2020). Default settings in DIA-NN were used except that Match Between Runs (MBR) was also selected. In addition, to increase the depth of our proteome analysis, an in silico generated PROSIT library^24^ of the annotated human proteome was generated within the software and used to directly interrogate the DIA data. DIA-NN aggregates peptide level quantifications to the protein level. The resulting CSV was pivoted within R and further analysed in Perseus^54^. Protein abundances were Log_2_ transformed, and proteins filtered for a minimum of three valid values per sample. Missing values were imputed following a normal distribution using the default values within Perseus. The dataset was then normalised using median abundances. The resulting spreadsheet was exported for further analysis and data visualisation in the R environment.

### Differential protein and transcript expression, upset plots, and gene ontology analysis

Limma Bioconductor R package was used to determine fold changes between groups. Volcano plots were generated by R package ggplot2^55^. UpsetR package^56^ was used to visualise and determine the numbers of unique genes in each sample set as well as intersecting proteins and their identities between datasets. Gene ontology analysis was then performed on the intersection proteins to determine the biological processes, molecular function, and cellular components and pathways involved in CKD using the Gene Ontology Database for Annotation, Visualization, and Integrated Discovery (DAVID) Bioinformatics Resource (v2022q3). Rat protein accession numbers were converted to gene names and imported into the database. Rivigo web server (http://revigo.irb.hr/) was subsequently employed to reduce the complexity and redundancy of the GO annotations. TreeMap R scripts generated in Rivigo were exported, and figures generated in R Studio after modification of the scripts to include -log_10_ *p*- values on the maps.

## Supplementary Information

Supplementary Information 1.

Supplementary Information 2.

Supplementary Information 3.

## Data Availability

The mass spectrometry proteomics data generated during the current study is available in ProteomeXchange Consortium via the PRIDE^57^ partner repository with the dataset identifier PXD048564. The transcriptomics data discussed in this publication have been deposited in NCBI Gene Expression Omnibus^58,59^ and are accessible through GEO Series accession numbers (https://www.ncbi.nlm.nih.gov/geo/query/acc.cgi?acc=GSExxx). The following accession numbers have been assigned to the data: GSE253009 (encompasses both study 1 and 2 kidney datasets), GSE253008 (subseries kidney study 1 dataset) and GSE253007 (subseries kidney study 2 dataset). All data analysed during this study are included in this published article (and its Supplementary Information files).
