# Supplementary material for: Identification of mechanistic CKD biomarkers in a rat SNx kidney fibrosis model by transcriptomics and proteomics detectable in biofluids
Source: Sci Rep. 2025 Apr 2;15:11200. doi: 10.1038/s41598-025-93894-6 (PMC11962143; doi:10.1038/s41598-025-93894-6)

Identification of mechanistic CKD biomarkers in a rat SNx kidney fibrosis model by transcriptomics and proteomics detectable in biofluids

Karin Barnouin, Elisa Tonoli, Clare Coveney, John Atkinson, Margarida Sancho, Andrew Skelton, David J Boocock, Linghong Huang, Joseph Shephard, Tim Johnson, Elisabetta AM Verderio, and Breda Twomey

Supplementary Methods

Subtotal Nephrectomy rat model generation

Animals were housed in open-top cages, in a specific-pathogen-free facility according to the Federation of European Laboratory Animal Science Associations recommendations. Typically, animals were housed in groups of four or five. Food (RM1; SAFE, Paris, France) and water (untreated mains tap water) were available ad libitum, and the light cycle was 12/12 hours light and dark. Room temperature and humidity were 21°C and 45%, respectively. Nest building material was provided, and all cages contained enrichment such as a red translucent house, a wheel and a tunnel.

Prior to surgery, male Wistar rats (200-300g) were acclimatised to the facility for one week. They were then dosed with pre-operative analgesia (0.01mg/kg Buprenorphine) and anaesthetised with isoflurane. The left flank was then shaved, the animals dosed with pre-operative carprofen (5mg/kg) and the animals transferred to the surgery suite. The animals were placed on an isoflurane nose-cone, covered with a sterile drape, and eye ointment was applied to prevent drying. The surgical site was cleaned and disinfected, and an incision made through the skin, and then blunt dissection was used to part the muscle later. The left kidney was decapsulated and the renal blood vessels clamped. The poles of the kidney were ligated using 3-0 mersilk sutures, and then excised using a scalpel. 3M tissue glue was applied to the cut ends. The clamp was then removed, and the kidney replaced into the abdomen. The muscle was sutured using 5-0 Vicryl in a continuous pattern, and the skin was sutured using 5-0 Vicryl in a sub-cuticular continuous pattern. Local anaesthetic (marcain (0.5mg/kg) was applied to the surgical site. Animals were allowed to recover on a warming rack and monitored for welfare. Animals received post-operative analgesia for 4 days.

One week later, the animals were prepared for surgery as above, and the right kidney decapsulated and clamped. The right renal blood vessels were ligated using 3-0 mersilk, and the entire right kidney removed. Animals were sutured as above, and allowed to recover as above, with post-operative analgesia for 4 days.

Animals were tail-pricked or bled (30 µL) for twice-weekly serum creatinine measurements and were placed in metabolic cages bi-weekly for proteinuria measurements. The animals were allocated to a study group in a random manner using a random number generator once they reached the threshold of 2-fold increase in serum creatinine and 3-5x fold increase in proteinuria compared to non-operated sham control. Animals were anaesthetised, bled by cardiac puncture, terminated by cervical dislocation, death confirmed, and the kidney remnant removed, cut into quarters, and either fixed in formalin or snap frozen in liquid nitrogen.

Analysis was performed in a blinded manner, with the analyst and the interpreting researcher unaware of which animals were sham or subtotal nephrectomy-operated.

UUO mouse model generation

UUO surgery was performed on male C57Bl6/J mice of between 20-30g body weight.

Prior to surgery, all animals are dosed with 100 µL pre-operative Buprenorphine (0.05mg/kg Mice / 0.01mg/kg Rats). Mice are anaesthetised with isoflurane and a small patch of skin shaved just below the ribs on the left flank. Mice are then dosed with pre-operative carprofen (5mg/kg). The mouse is laid on the operating table on a sterile drape under isoflurane anaesthesia. The surgical site is disinfected with chlorhexidine and the animal covered by a sterile drape. A small incision is made below the ribcage, parallel to the spine, the muscle is exposed by blunt dissection and a small incision made. The opening in the muscle is widened using blunt dissection. The ureter is located and ligated using 2x 3-0 Mersilk (Ethicon). The ureter is replaced, and the muscle sutured using 5-0 Vicryl (Ethicon). The skin is then sutured in a sub-cuticular pattern with 5-0 Vicryl (Ethicon). The wound is cleaned with chlorhexidine and levobupivacaine administered to the site (0.5mg/kg). Surgical duration is approximately 15-20 minutes, and animals are monitored throughout for surgical anaesthetic plane. Animals were returned to their home cage and received post-operative analgesia for 4 days (Buprenorphine and Carprofen at the doses above). All mice were housed under specific pathogen-free conditions and maintained on a 12- hour light/dark cycle with free access to food and water.

Animals were monitored daily for welfare scoring and body weight. At Day 21 animals were terminated by a schedule 1 method and tissues harvested.

Adriamycin mice model generation:

The Adriamycin model was performed on male BalbC mice of between 20-30g body weight.

A dose of 10.5mg/kg Adriamycin was administered via the tail vein of unanaesthetised mice. Animals were returned to their home cage and monitored daily for welfare scoring and body weight. Urine was collected prior to termination using a metabolic cage. Termination was performed by a schedule 1 method at day 14 and tissues collected. All mice were housed under specific pathogen-free conditions and maintained on a 12- hour light/dark cycle with free access to food and water. 24-hour urine samples were collected prior to injection of Adriamycin and termination, so there were 2 urine collections per animal.

Collagen Picrosirius Red (PSR) Staining

Kidney samples were harvested and fixed in 10% formalin for 24 hours. The samples were coded for blinding and placed in a labelled cassette, which entails further formalin fixation, dehydration in increasing concentrations of alcohol, clearing in xylene and impregnation with paraffin wax. After this, samples were orientated in paraffin wax with the cassette placed on top of the embedding moulds. The kidney samples were sectioned at 3 µm using a Leica Histocore multicut. These sections were floated onto a warm water bath and were placed onto labelled Superfrost Plus Gold slides for staining. The slides were de-waxed and hydrated.

Nuclei were stained with Weigert's haematoxylin for 8 minutes, and then the slides washed for 10 minutes in running tap water. The slides were stained in picro-sirius red solution for one hour, and then washed in two changes of acidified water. Water was removed, and the slides dehydrated in 100% alcohol. The slides were then cleared with xylene, and coverslips applied. Whole slides were then scanned using an OlyVIA slide scanner (Olympus) and given codes for the blinding of the image analysis.

Image Analysis

Whole slide scans were uploaded to VisioPharm Image analysis software. In a blinded manner, the cortices of the kidneys were manually annotated, excluding the medulla from analysis. Thresholds were set for detecting red-stained collagen and the software allowed to run this detection over the entire cortex of each kidney. Data were outputted as the percentage area of the renal cortex that stained positive for collagen / PSR staining.

Supplementary Figure 1

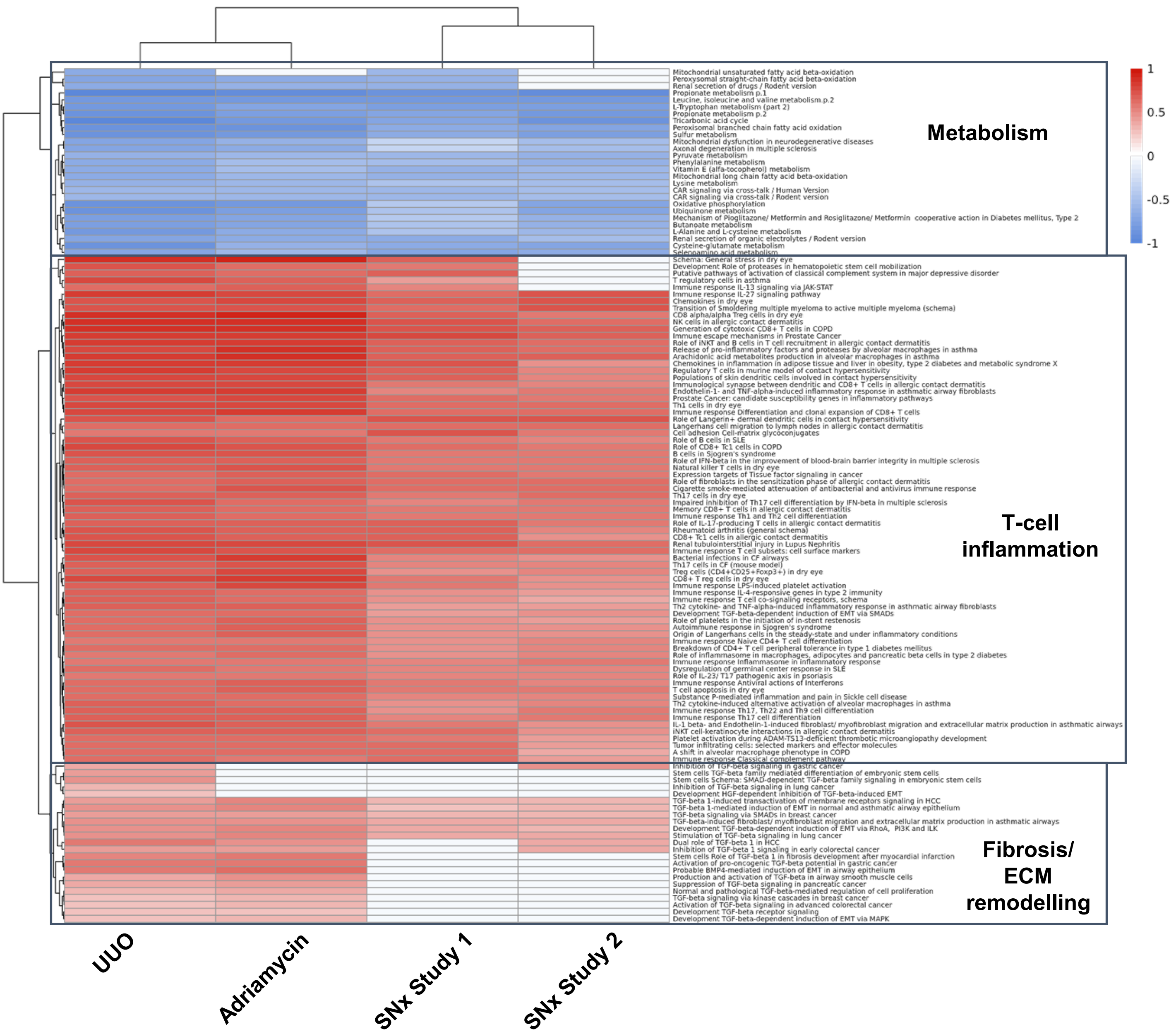

Supplementary figure 1: Comparison of transcriptomics profiles of rat SNx with other CKD models.

Supplementary Figure 2

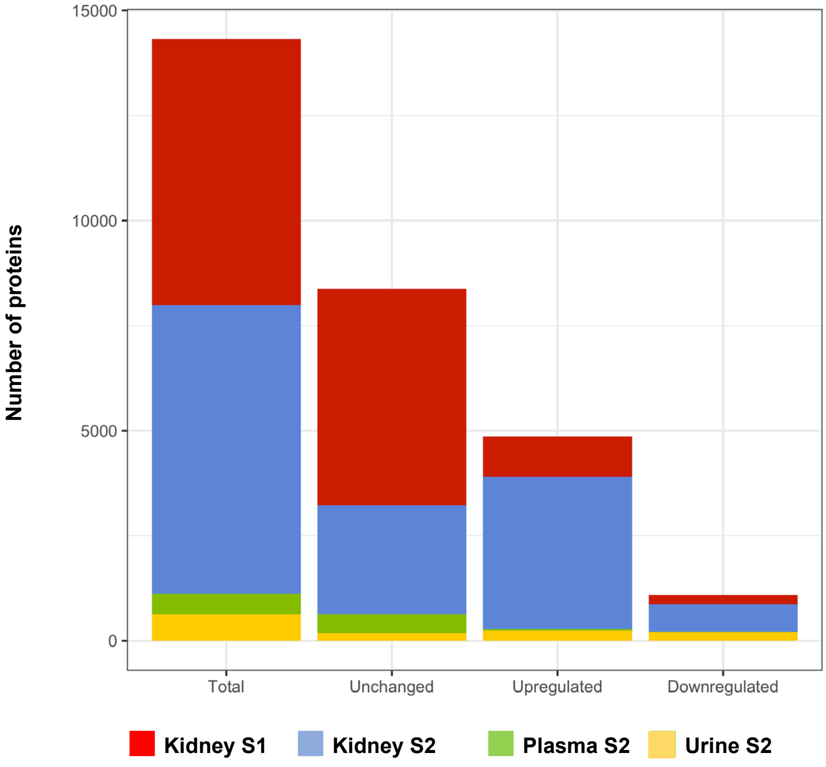

Supplementary figure 2: Summary of protein counts

Supplementary Figure 3

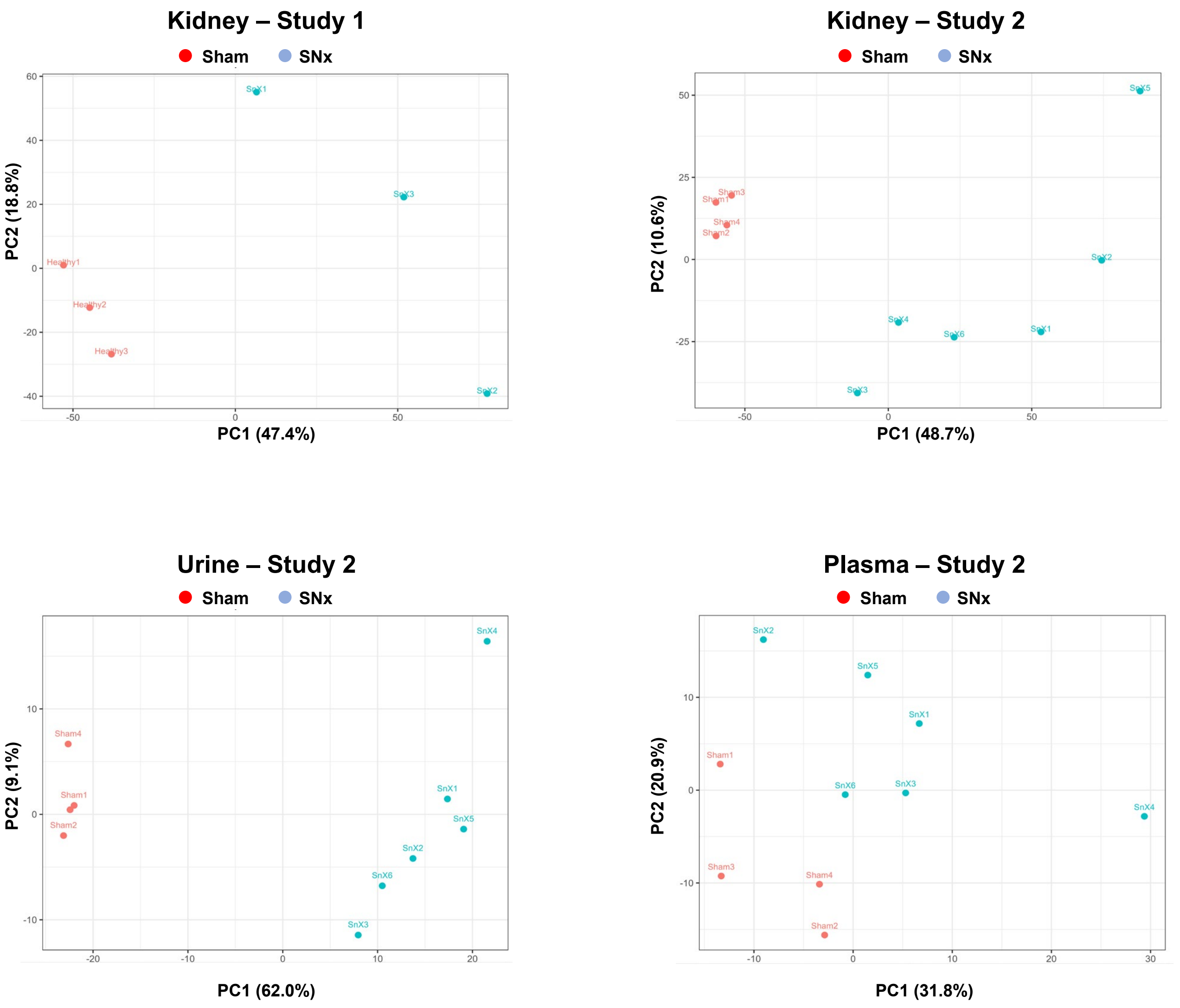

Supplementary Figure 3: PCA analysis of proteomic data.

Supplementary Figure 4

Supplementary figure 4: TreeMaps Biological Processes GO enriched in unsupervised cluster analysis – DAVID – KEGG of proteomic data

Supplementary Figure 4a

a

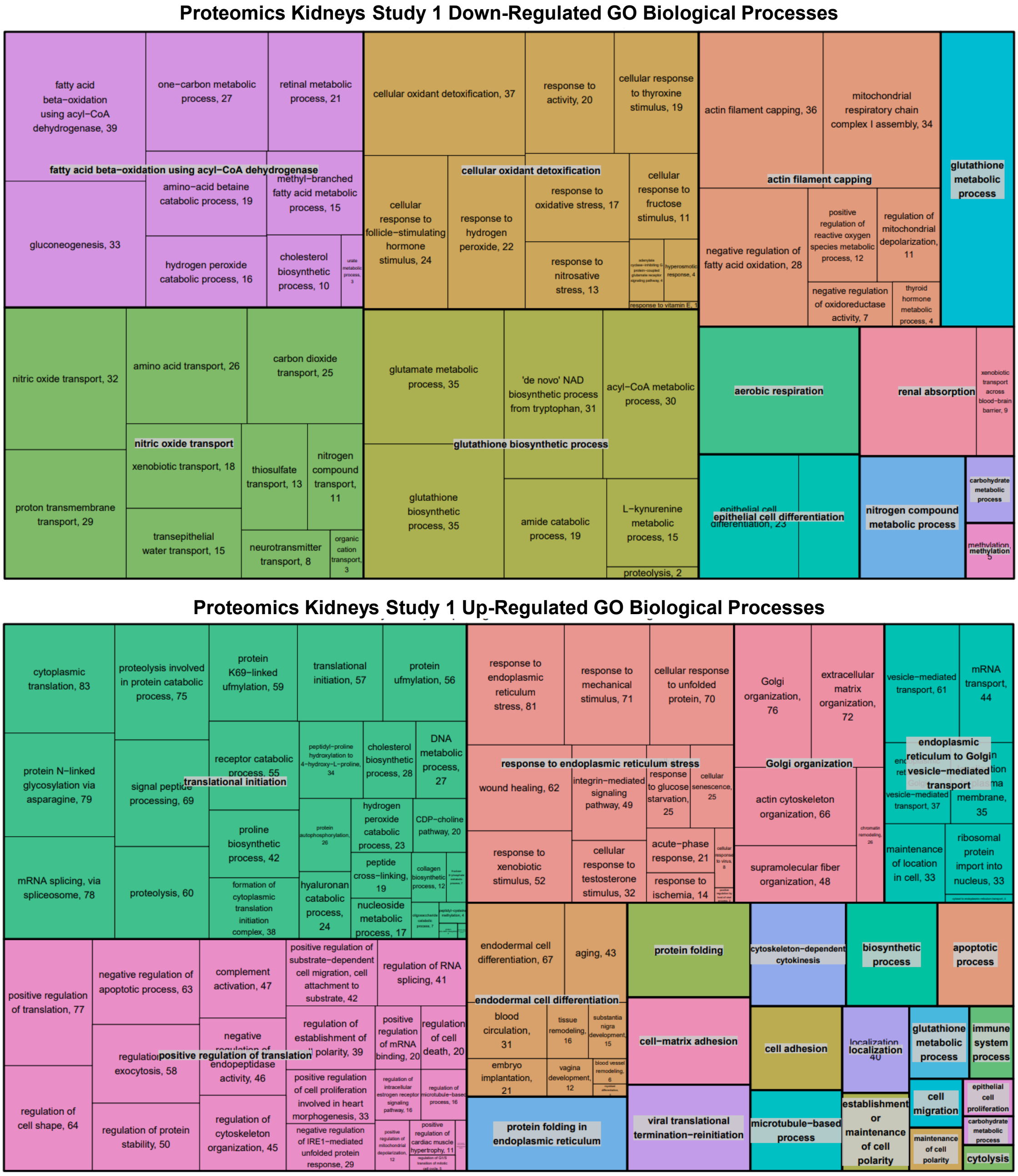

Supplementary figure 4a: TreeMaps of down- and up-regulated proteins enriched in Kidney Study 1

Supplementary Figure 4b

b

Proteomics Kidneys Study 2 Down-Regulated GO Biological Processes

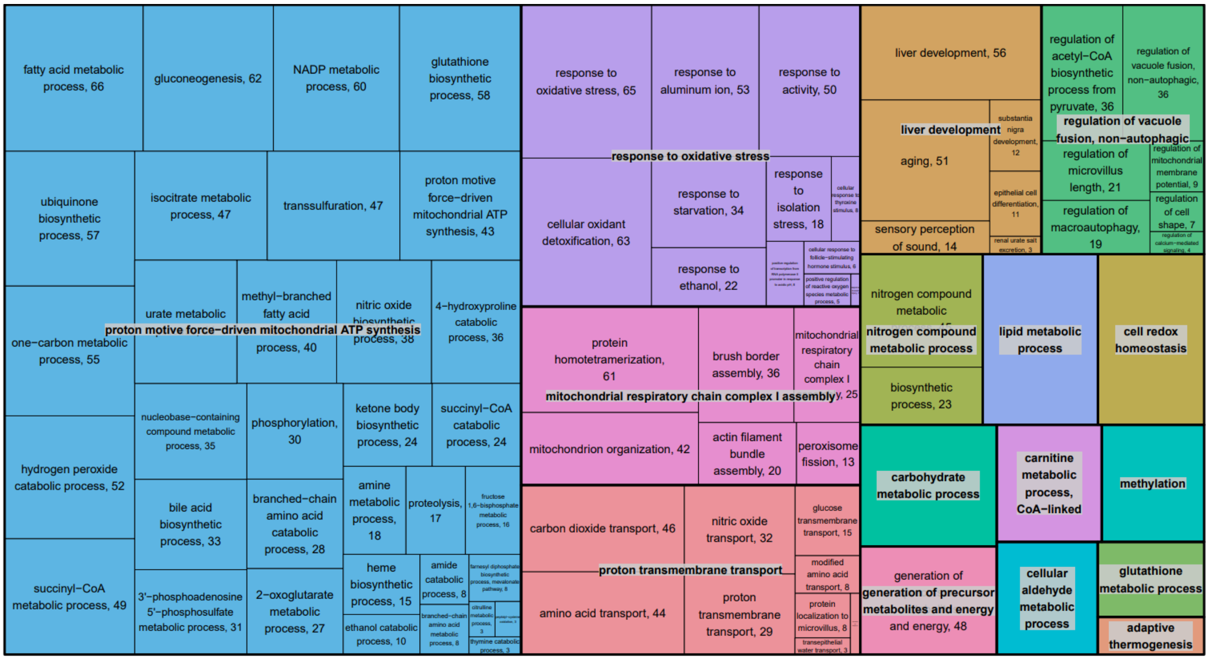

Proteomics Kidneys Study 2 Up-Regulated GO Biological Processes

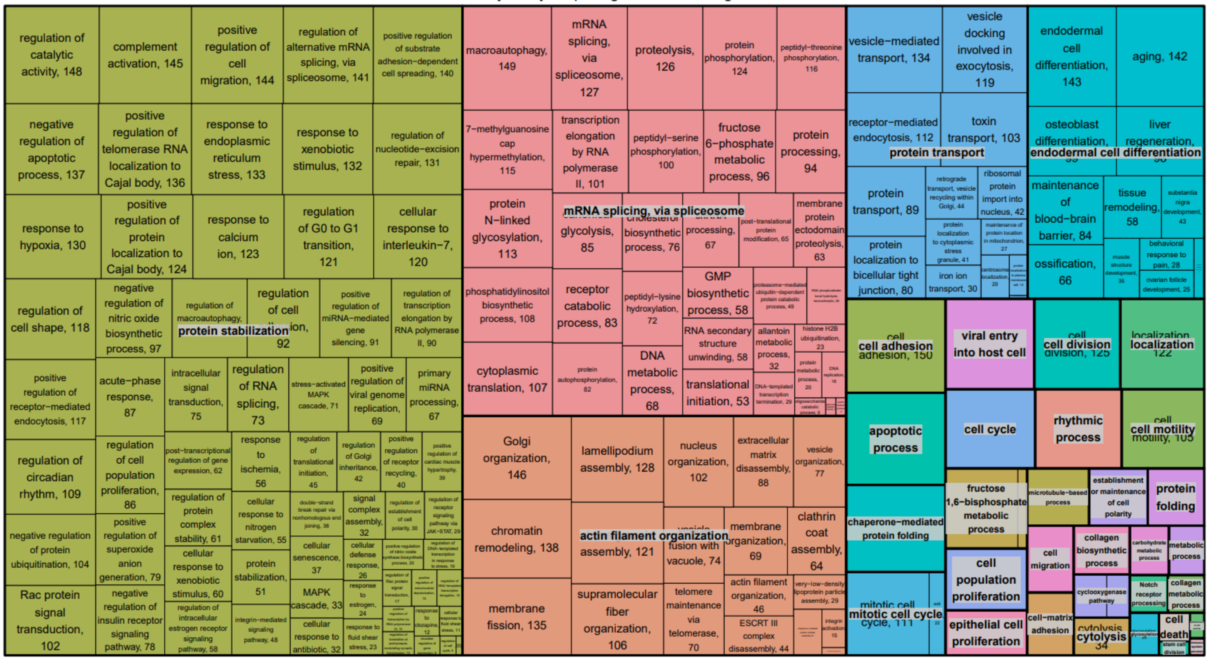

Supplementary figure 4b: TreeMaps of down- and up-regulated proteins enriched in Kidney Study 2

Supplementary Figure 4c

c

Proteomics Urine Study 2 Down-Regulated GO Biological Processes

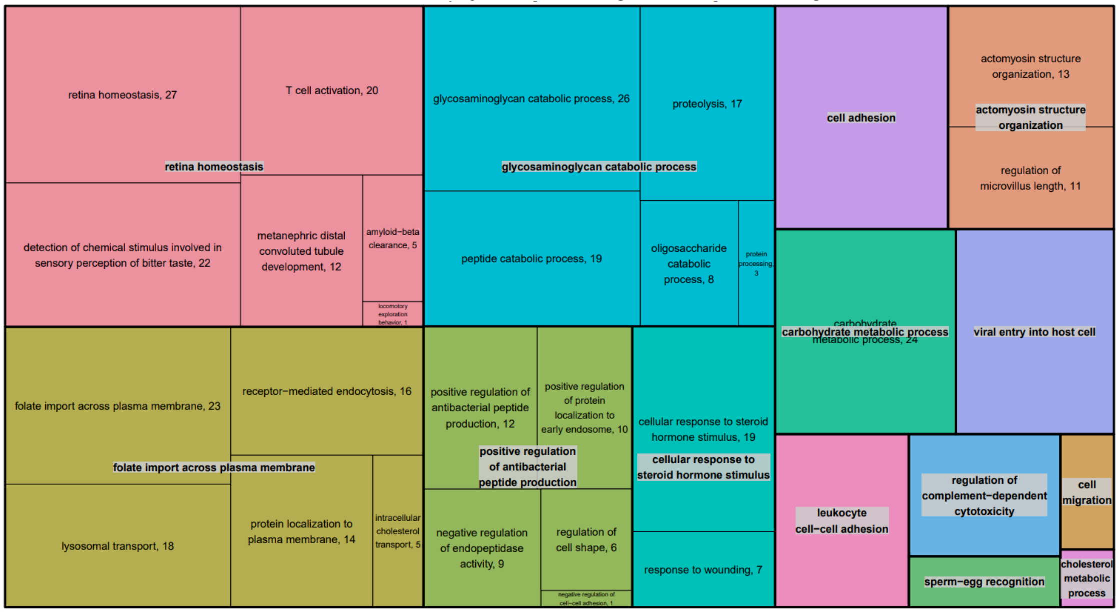

Proteomics Urine Study 2 Up-Regulated GO Biological Processes

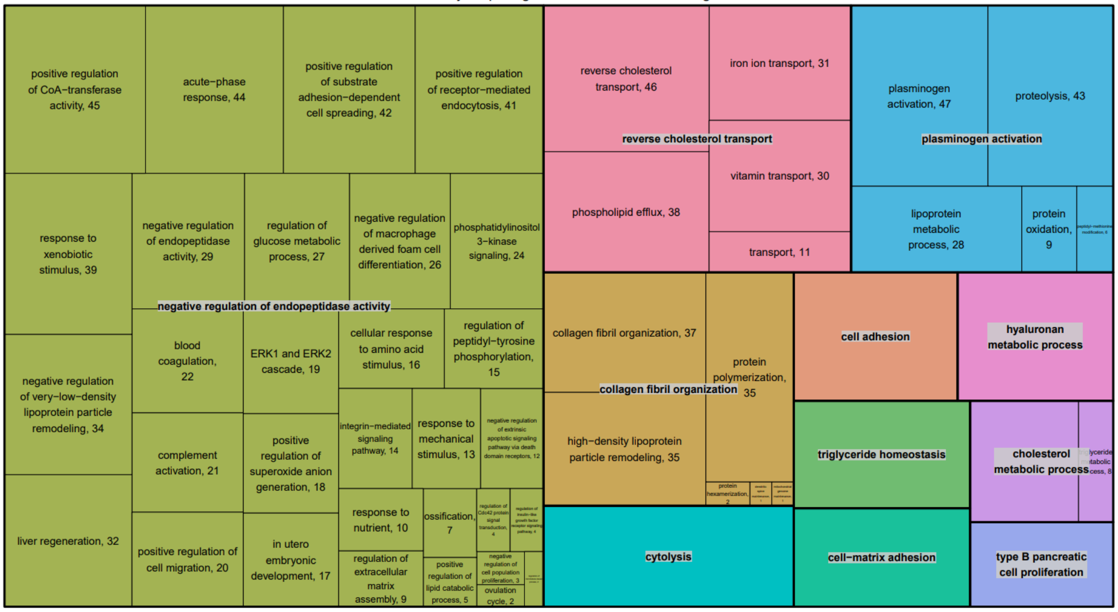

Proteomics Plasma Study 2 Up-Regulated GO Biological Processes

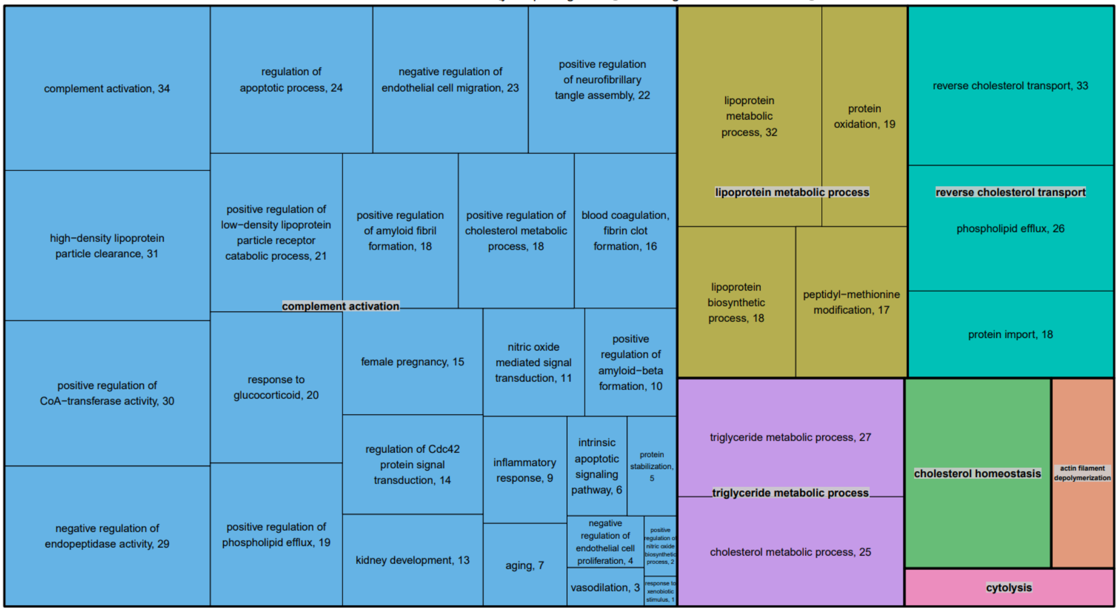

Supplementary figure 4c: TreeMaps of down- and up-regulated proteins in Study 2 Plasma and Urine

Supplementary Figure 5

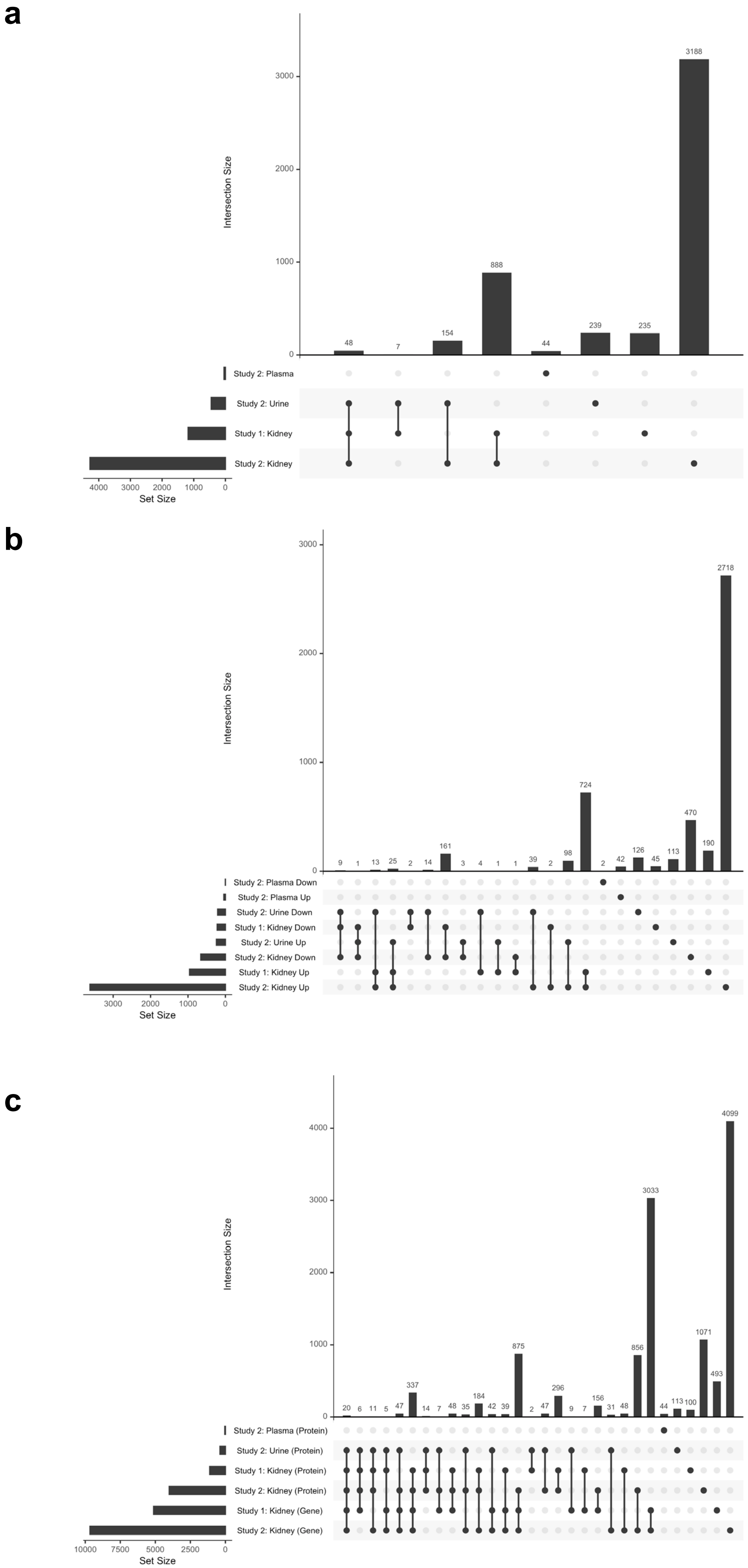

Supplementary figure 5: Upset plots comparing different tissues or analyses. a) Proteomics intersections in Kidneys, Plasma and Urine (total numbers). **b)** Proteomics intersections in Kidneys, Plasma and Urine (significantly changed proteins). **c)** Proteomics and transcriptomics intersections in Kidneys, Plasma and Urine (total numbers).

Supplementary Figure 6

Supplementary Figure 6: TreeMaps GO (Biological processes, Molecular Function and Cellular Component) enriched in unsupervised cluster analysis – DAVID in proteomic and transcriptomic datasets. a) GO enriched in up-regulated proteins in both Kidney Studies 1 and 2. **b)** GO enriched in down-regulated proteins in both kidney Studies 1 and 2. **c)** GO enriched in up-regulated proteins in both Study 2 kidney and urine. **d)** GO enriched in down-regulated proteins in both Study 2 kidney and urine. **e)** GO enriched in down-regulated proteins and genes in both Study 1 kidney proteomic and transcriptomics datasets. **f)** GO enriched in down-regulated proteins and genes in both Study 2 kidney proteomic and transcriptomics datasets. **g)** GO enriched in down-regulated proteins and genes in both Studies 1 and 2 kidney proteomic and transcriptomics datasets.

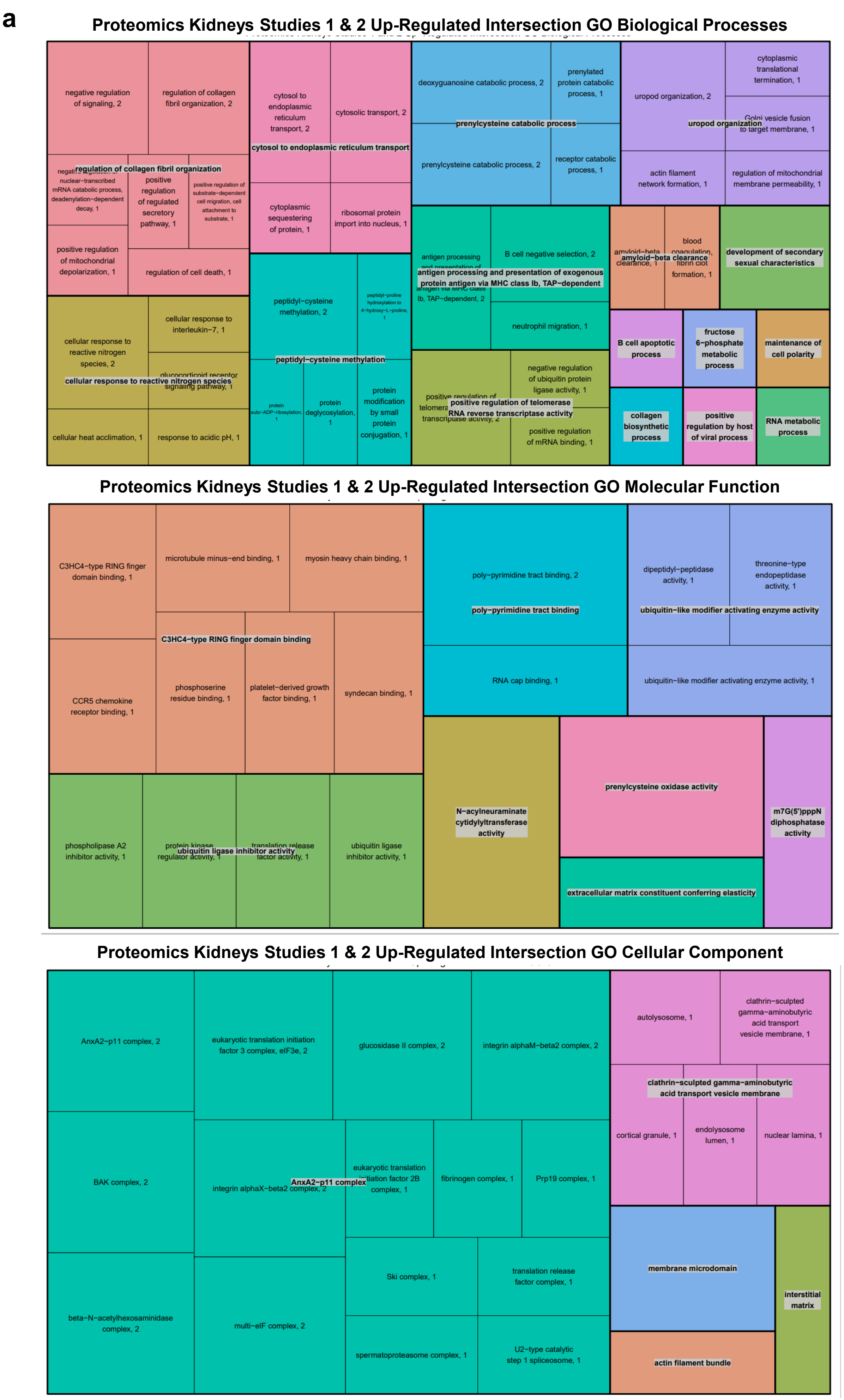
a
Proteomics Kidneys Studies 1 & 2 Up-Regulated Intersection GO Biological Processes
negative regulation of signaling, 2
regulation of collagen fibril organization, 2
regulation of collagen fibril organization
nuclear−transcribed mRNA catabolic process, deadenylation−dependent decay, 1
positive regulation of regulated secretory pathway, 1
positive regulation of substrate−dependent cell migration, cell attachment to substrate, 1
positive regulation of mitochondrial depolarization, 1
regulation of cell death, 1
cytosol to endoplasmic reticulum transport, 2
cytosolic transport, 2
cytosol to endoplasmic reticulum transport
cytoplasmic sequestering of protein, 1
ribosomal protein import into nucleus, 1
deoxyguanosine catabolic process, 2
prenylated protein catabolic process, 1
prenylcysteine catabolic process
prenylcysteine catabolic process, 2
receptor catabolic process, 1
uropod organization, 2
cytoplasmic translational termination, 1
Golgi vesicle fusion to target membrane, 1
uropod organization
actin filament network formation, 1
regulation of mitochondrial membrane permeability, 1
cellular response to reactive nitrogen species, 2
cellular response to interleukin−7, 1
cellular response to reactive nitrogen species
glucocorticoid receptor signaling pathway, 1
cellular heat acclimation, 1
response to acidic pH, 1
peptidyl−cysteine methylation, 2
peptidyl−proline hydroxylation to 4−hydroxy−L−proline, 1
peptidyl−cysteine methylation
protein auto−ADP−ribosylation, 1
protein deglycosylation, 1
protein modification by small protein conjugation, 1
antigen processing
B cell negative selection, 2
antigen processing and presentation of exogenous protein antigen via MHC class Ib, TAP−dependent
Ib, TAP−dependent, 2
neutrophil migration, 1
positive regulation of telomerase RNA reverse transcriptase activity
negative regulation of ubiquitin protein ligase activity, 1
positive regulation of mRNA binding, 1
amyloid−beta clearance
blood coagulation, fibrin clot formation, 1
development of secondary sexual characteristics
B cell apoptotic process
fructose 6−phosphate metabolic process
maintenance of cell polarity
collagen biosynthetic process
positive regulation by host of viral process
RNA metabolic process
Proteomics Kidneys Studies 1 & 2 Up-Regulated Intersection GO Molecular Function
C3HC4−type RING finger domain binding, 1
microtubule minus−end binding, 1
myosin heavy chain binding, 1
C3HC4−type RING finger domain binding
CCR5 chemokine receptor binding, 1
phosphoserine residue binding, 1
platelet−derived growth factor binding, 1
syndecan binding, 1
poly−pyrimidine tract binding, 2
poly−pyrimidine tract binding
RNA cap binding, 1
dipeptidyl−peptidase activity, 1
threonine−type endopeptidase activity, 1
ubiquitin−like modifier activating enzyme activity
ubiquitin−like modifier activating enzyme activity, 1
phospholipase A2 inhibitor activity, 1
protein kinase regulator activity, 1
ubiquitin ligase inhibitor activity
translation release factor activity, 1
ubiquitin ligase inhibitor activity, 1
N−acylneuraminate cytidylyltransferase activity
prenylcysteine oxidase activity
extracellular matrix constituent conferring elasticity
m7G(5')pppN diphosphatase activity
Proteomics Kidneys Studies 1 & 2 Up-Regulated Intersection GO Cellular Component
AnxA2−p11 complex, 2
eukaryotic translation initiation factor 3 complex, eIF3e, 2
glucosidase II complex, 2
integrin alphaM−beta2 complex, 2
BAK complex, 2
integrin alphaX−beta2 complex, 2
AnxA2−p11 complex
eukaryotic translation initiation factor 2B complex, 1
fibrinogen complex, 1
Prp19 complex, 1
beta−N−acetylhexosaminidase complex, 2
multi−eIF complex, 2
Ski complex, 1
translation release factor complex, 1
spermatoproteasome complex, 1
U2−type catalytic step 1 spliceosome, 1
autolysosome, 1
clathrin−sculpted gamma−aminobutyric acid transport vesicle membrane, 1
clathrin−sculpted gamma−aminobutyric acid transport vesicle membrane
cortical granule, 1
endolysosome lumen, 1
nuclear lamina, 1
membrane microdomain
interstitial matrix
actin filament bundle

b

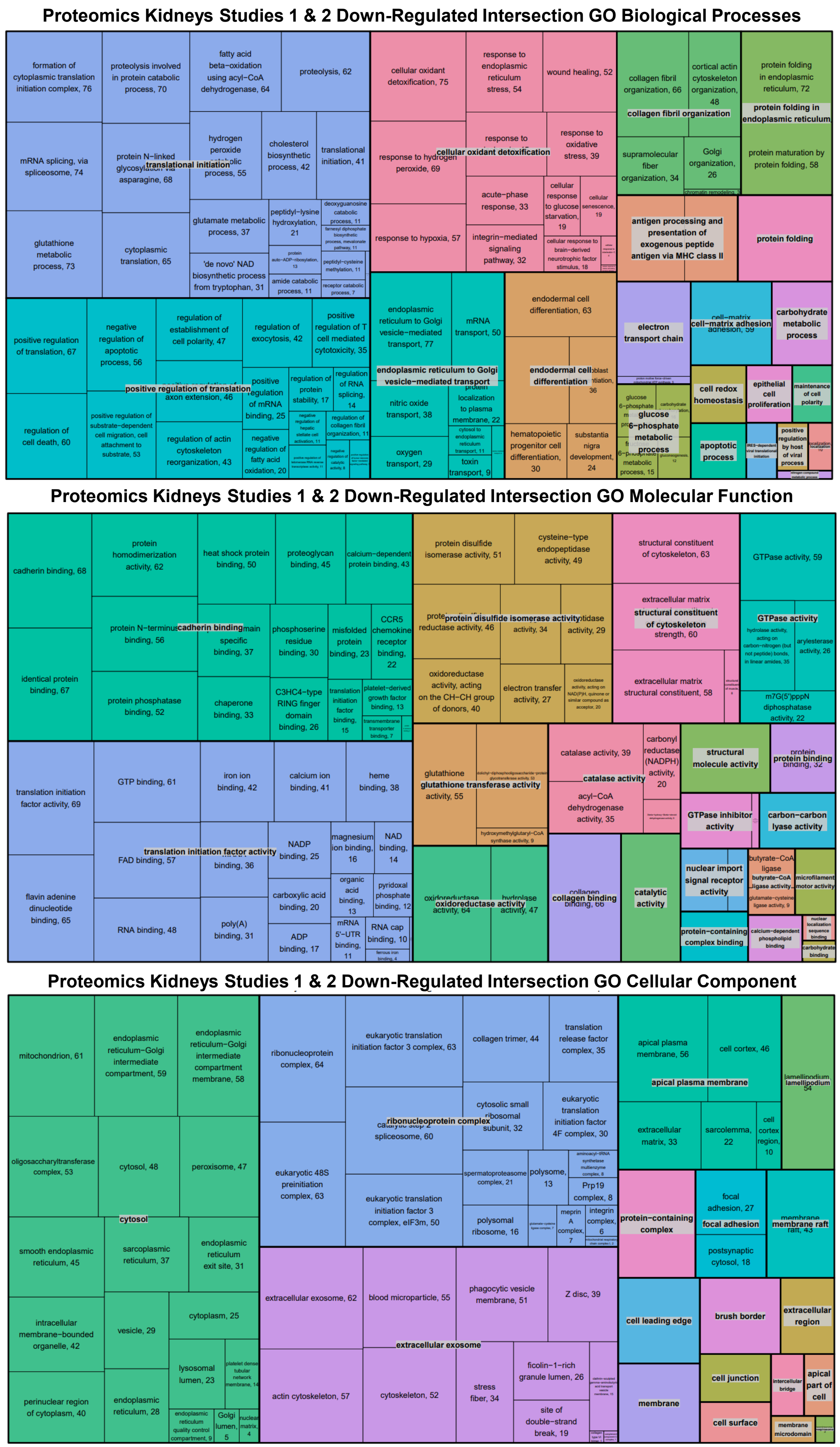

Proteomics Kidneys Studies 1 & 2 Down-Regulated Intersection GO Biological Processes
formation of cytoplasmic translation initiation complex, 76
proteolysis involved in protein catabolic process, 70
fatty acid beta−oxidation using acyl−CoA dehydrogenase, 64
proteolysis, 62
mRNA splicing, via spliceosome, 74
protein N−linked glycosylation via asparagine, 68
translational initiation
hydrogen peroxide catabolic process, 55
cholesterol biosynthetic process, 42
translational initiation, 41
glutathione metabolic process, 73
cytoplasmic translation, 65
glutamate metabolic process, 37
peptidyl−lysine hydroxylation, 21
deoxyguanosine catabolic process, 11
'de novo' NAD biosynthetic process from tryptophan, 31
amide catabolic process, 11
peptidyl−cysteine methylation, 11
receptor catabolic process, 7
cellular oxidant detoxification, 75
response to endoplasmic reticulum stress, 54
wound healing, 52
response to hydrogen peroxide, 69
cellular oxidant detoxification
response to oxidative stress, 39
acute−phase response, 33
response to hypoxia, 57
integrin−mediated signaling pathway, 32
cellular response to glucose starvation, 19
cellular senescence, 19
cellular response to brain−derived neurotrophic factor stimulus, 18
collagen fibril organization, 66
cortical actin cytoskeleton organization, 48
protein folding in endoplasmic reticulum, 72
collagen fibril organization
protein folding in endoplasmic reticulum
supramolecular fiber organization, 34
Golgi organization, 26
protein maturation by protein folding, 58
chromatin remodeling, 3
antigen processing and presentation of exogenous peptide antigen via MHC class II
protein folding
positive regulation of translation, 67
negative regulation of apoptotic process, 56
regulation of establishment of cell polarity, 47
regulation of exocytosis, 42
positive regulation of T cell mediated cytotoxicity, 35
positive regulation of translation
axon extension, 46
positive regulation of mRNA binding, 25
regulation of protein stability, 17
regulation of RNA splicing, 14
regulation of cell death, 60
positive regulation of substrate−dependent cell migration, cell attachment to substrate, 53
regulation of actin cytoskeleton reorganization, 43
negative regulation of fatty acid oxidation, 20
regulation of collagen fibril organization, 11
endoplasmic reticulum to Golgi vesicle−mediated transport, 77
mRNA transport, 50
endoplasmic reticulum to Golgi vesicle−mediated transport
nitric oxide transport, 38
protein localization to plasma membrane, 22
oxygen transport, 29
cytosol to endoplasmic reticulum transport, 11
toxin transport, 9
endodermal cell differentiation, 63
endodermal cell differentiation
hematopoietic progenitor cell differentiation, 30
substantia nigra development, 24
electron transport chain
cell−matrix adhesion
cell−matrix adhesion, 59
carbohydrate metabolic process
cell redox homeostasis
epithelial cell proliferation
maintenance of cell polarity
glucose 6−phosphate metabolic process
glucose 6−phosphate metabolic process, 15
apoptotic process
positive regulation by host of viral process
Proteomics Kidneys Studies 1 & 2 Down-Regulated Intersection GO Molecular Function
cadherin binding, 68
protein homodimerization activity, 62
heat shock protein binding, 50
proteoglycan binding, 45
calcium−dependent protein binding, 43
protein N−terminus binding, 56
cadherin binding
domain specific binding, 37
phosphoserine residue binding, 30
misfolded protein binding, 23
CCR5 chemokine receptor binding, 22
identical protein binding, 67
protein phosphatase binding, 52
chaperone binding, 33
C3HC4−type RING finger domain binding, 26
translation initiation factor binding, 15
platelet−derived growth factor binding, 13
transmembrane transporter binding, 7
protein disulfide isomerase activity, 51
cysteine−type endopeptidase activity, 49
protein disulfide isomerase activity
reductase activity, 46
activity, 34
activity, 29
oxidoreductase activity, acting on the CH−CH group of donors, 40
electron transfer activity, 27
structural constituent of cytoskeleton, 63
extracellular matrix structural constituent conferring tensile strength, 60
structural constituent of cytoskeleton
extracellular matrix structural constituent, 58
GTPase activity, 59
GTPase activity
hydrolase activity, acting on carbon−nitrogen (but not peptide) bonds, in linear amides, 35
arylesterase activity, 26
m7G(5')pppN diphosphatase activity, 22
translation initiation factor activity, 69
GTP binding, 61
iron ion binding, 42
calcium ion binding, 41
heme binding, 38
FAD binding, 57
translation initiation factor activity
binding, 36
NADP binding, 25
magnesium ion binding, 16
NAD binding, 14
flavin adenine dinucleotide binding, 65
RNA binding, 48
poly(A) binding, 31
carboxylic acid binding, 20
organic acid binding, 13
pyridoxal phosphate binding, 12
ADP binding, 17
mRNA 5'−UTR binding, 11
RNA cap binding, 10
glutathione transferase activity, 55
glutathione transferase activity
hydroxymethylglutaryl−CoA synthase activity, 9
catalase activity, 39
catalase activity
acyl−CoA dehydrogenase activity, 35
carbonyl reductase (NADPH) activity, 20
structural molecule activity
protein binding
protein binding, 32
GTPase inhibitor activity
carbon−carbon lyase activity
oxidoreductase activity
oxidoreductase activity, 64
hydrolase activity, 47
collagen binding
collagen binding, 66
catalytic activity
nuclear import signal receptor activity
butyrate−CoA ligase activity
microfilament motor activity
protein−containing complex binding
calcium−dependent phospholipid binding
carbohydrate binding
Proteomics Kidneys Studies 1 & 2 Down-Regulated Intersection GO Cellular Component
mitochondrion, 61
endoplasmic reticulum−Golgi intermediate compartment, 59
endoplasmic reticulum−Golgi intermediate compartment membrane, 58
oligosaccharyltransferase complex, 53
cytosol, 48
peroxisome, 47
cytosol
smooth endoplasmic reticulum, 45
sarcoplasmic reticulum, 37
endoplasmic reticulum exit site, 31
intracellular membrane−bounded organelle, 42
vesicle, 29
cytoplasm, 25
lysosomal lumen, 23
platelet dense tubular network membrane, 14
perinuclear region of cytoplasm, 40
endoplasmic reticulum, 28
endoplasmic reticulum quality control compartment, 9
Golgi lumen, 5
nuclear matrix, 4
ribonucleoprotein complex, 64
eukaryotic translation initiation factor 3 complex, 63
collagen trimer, 44
translation release factor complex, 35
ribonucleoprotein complex
cytosolic small ribosomal subunit, 32
eukaryotic translation initiation factor 4F complex, 30
spliceosome, 60
eukaryotic 48S preinitiation complex, 63
spermatoproteasome complex, 21
polysome, 13
Prp19 complex, 8
eukaryotic translation initiation factor 3 complex, eIF3m, 50
polysomal ribosome, 16
meprin A complex, 7
integrin complex, 6
extracellular exosome, 62
blood microparticle, 55
phagocytic vesicle membrane, 51
Z disc, 39
extracellular exosome
actin cytoskeleton, 57
cytoskeleton, 52
stress fiber, 34
ficolin−1−rich granule lumen, 26
site of double−strand break, 19
apical plasma membrane, 56
cell cortex, 46
apical plasma membrane
lamellipodium
lamellipodium, 54
extracellular matrix, 33
sarcolemma, 22
cell cortex region, 10
protein−containing complex
focal adhesion, 27
focal adhesion
membrane raft
membrane raft, 43
postsynaptic cytosol, 18
cell leading edge
brush border
extracellular region
cell junction
intercellular bridge
apical part of cell
membrane
cell surface
membrane microdomain

c

Proteomics Kidney & Urine Study 2 Up-Regulated Intersection GO Biological Processes

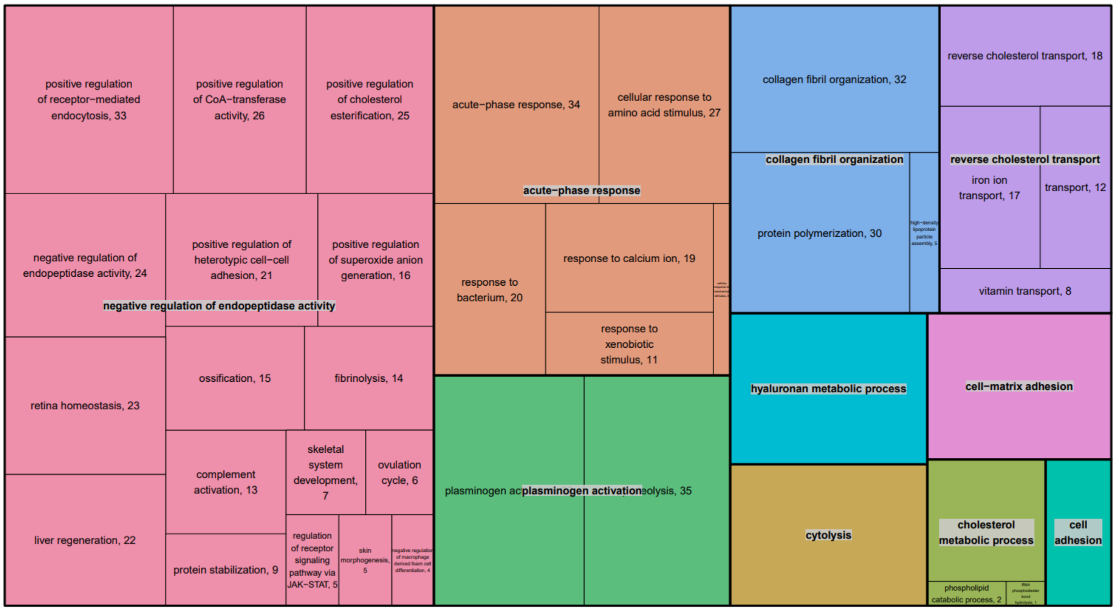

Proteomics Kidney & Urine Study 2 Up-Regulated Intersection GO Molecular Function

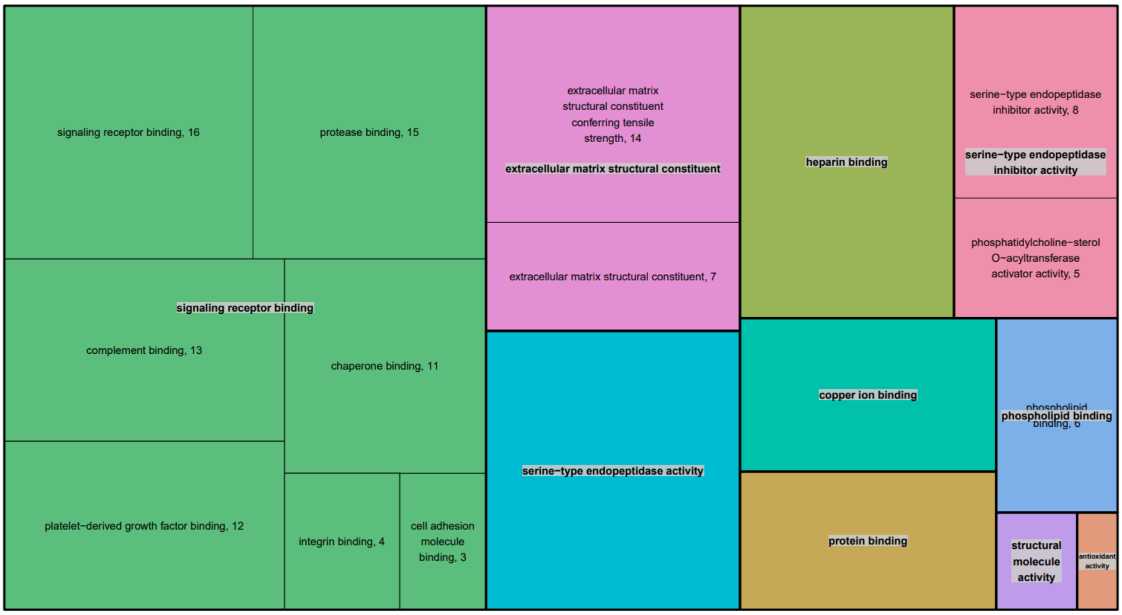

Proteomics Kidney & Urine Study 2 Up-Regulated Intersection GO Cellular Component

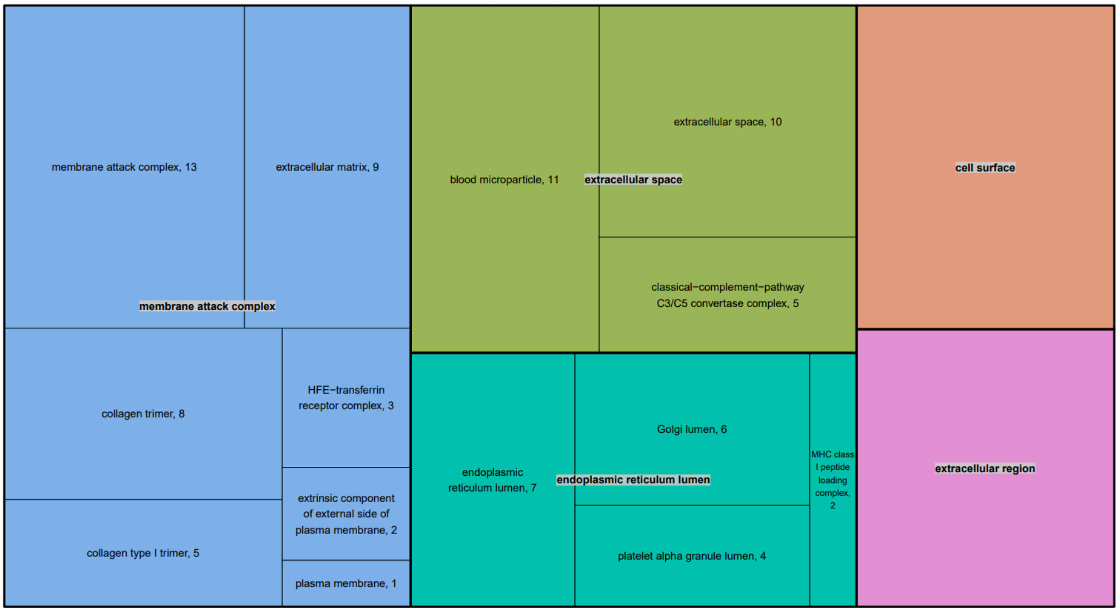

d

Proteomics Kidney & Urine Study 2 Down-Regulated Intersection GO Biological Processes

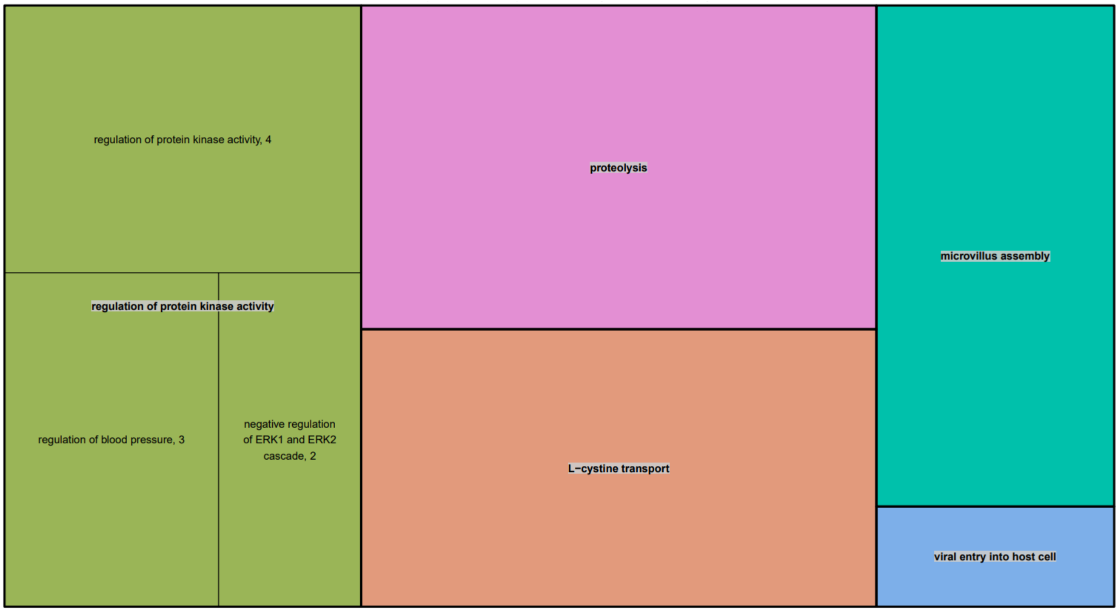

Proteomics Kidney & Urine Study 2 Down-Regulated Intersection GO Molecular Function

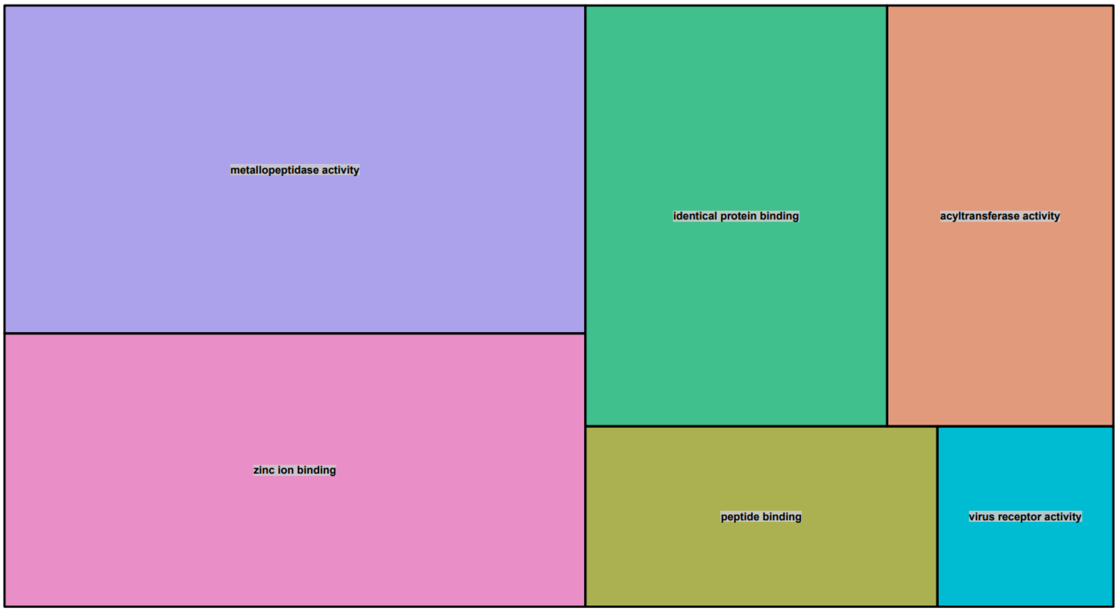

Proteomics Kidney & Urine Study 2 Down-Regulated Intersection GO Cellular Component

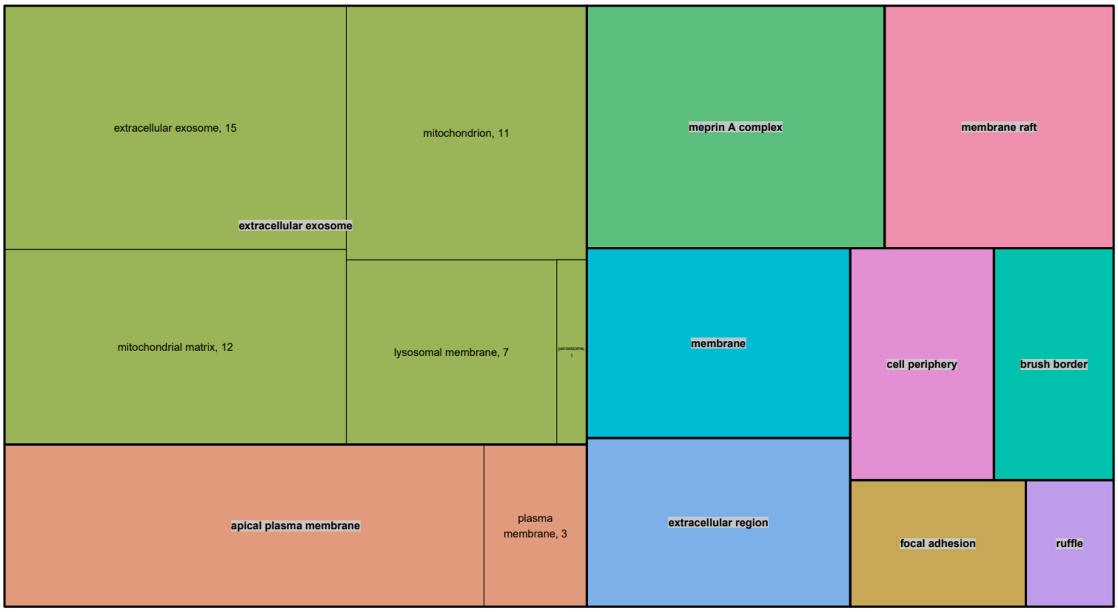

e

Kidney Study 1 Proteomics and Transcriptomics Down-Regulated Intersection GO Biological Processes

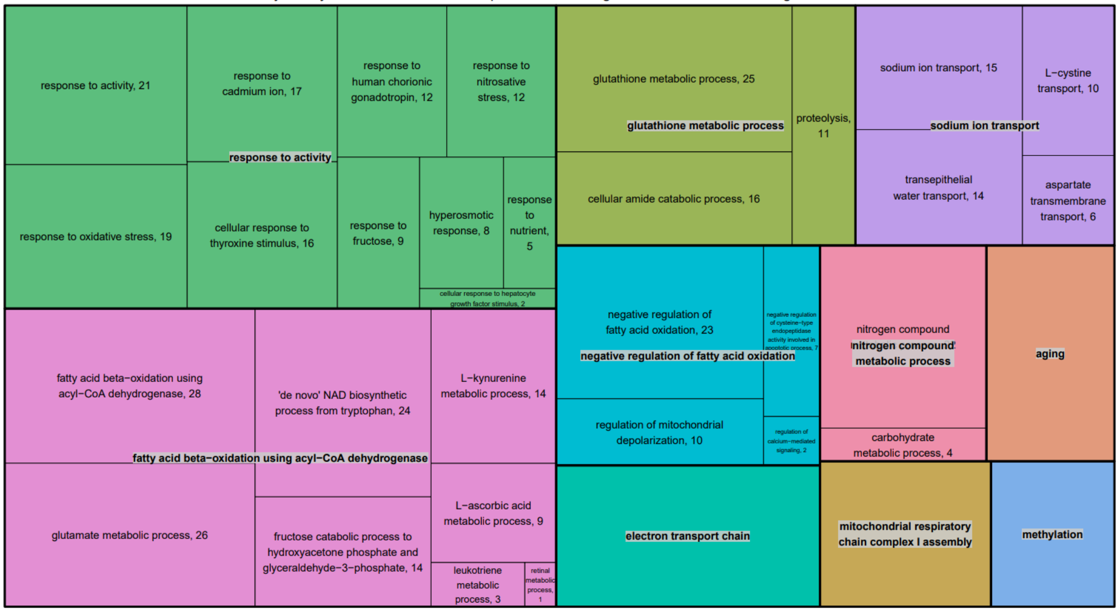

Kidney Study 1 Proteomics and Transcriptomics Down-Regulated Intersection GO Molecular Function

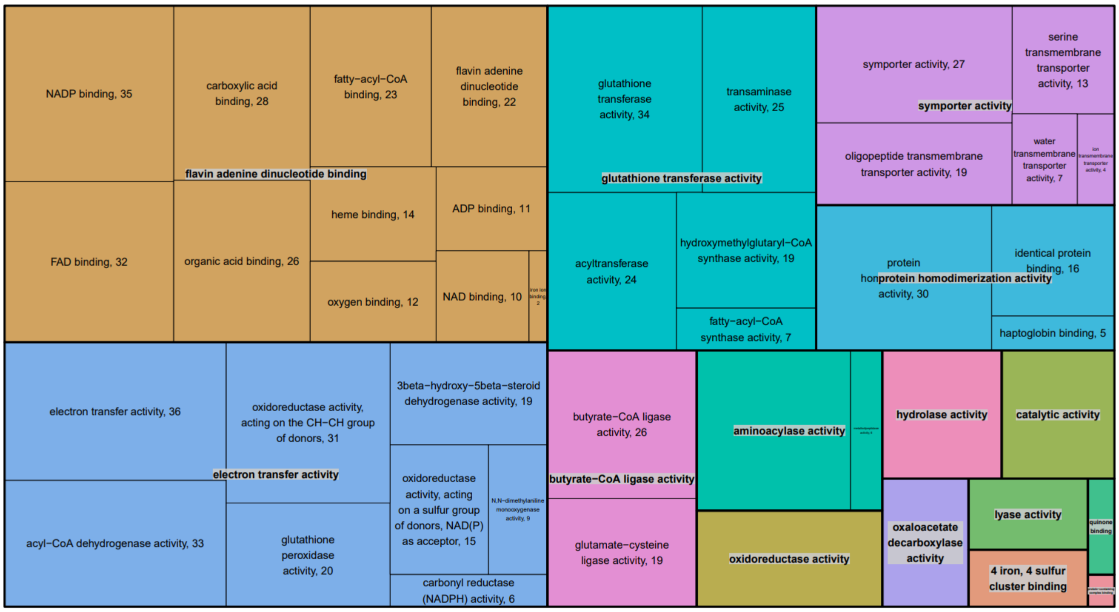

Kidney Study 1 Proteomics and Transcriptomics Down-Regulated Intersection GO Cellular Component

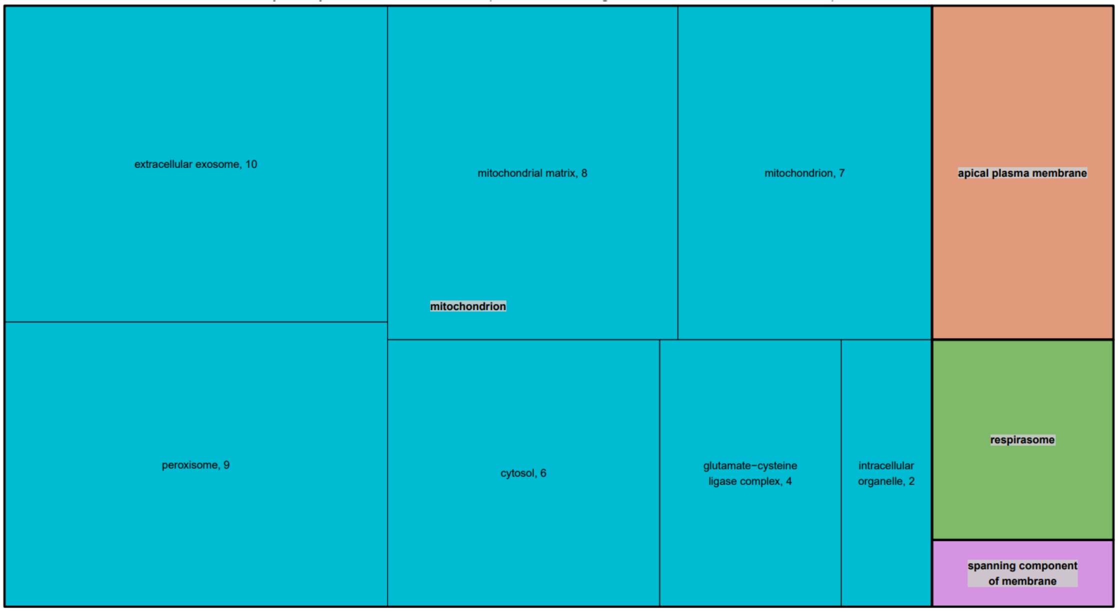

f

Kidney Study 2 Proteomics and Transcriptomics Down-Regulated Intersection GO Biological Processes

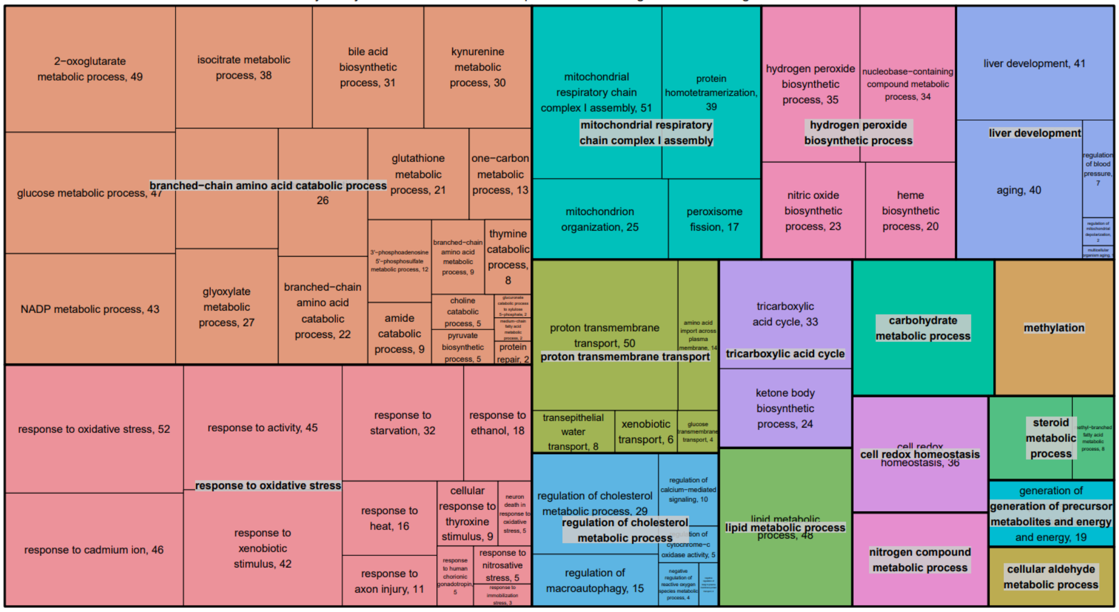

Kidney Study 2 Proteomics and Transcriptomics Down-Regulated Intersection GO Molecular Function

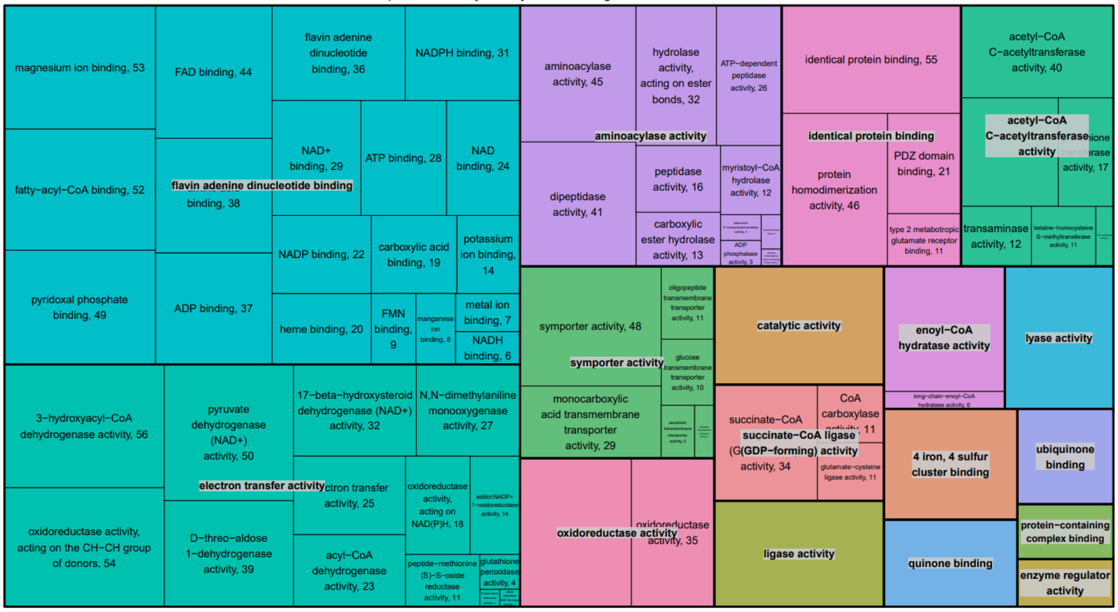

Kidney Study 2 Proteomics and Transcriptomics Down-Regulated Intersection GO Cellular Component

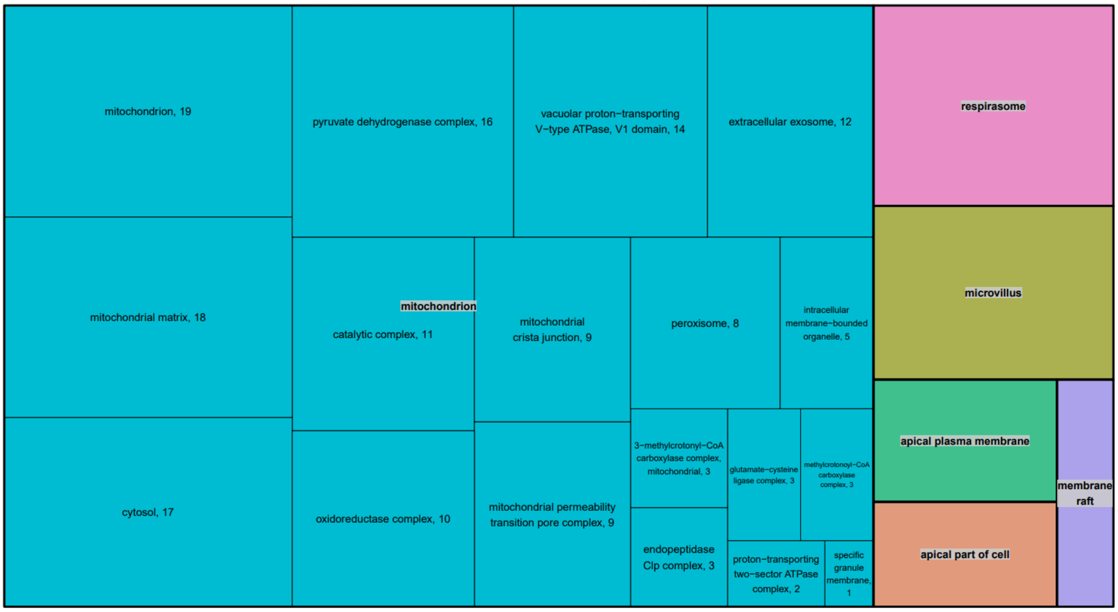

g

Proteomics and Transcriptomics Kidney Studies 1&2 Down-Regulated Intersection GO Biological Processes

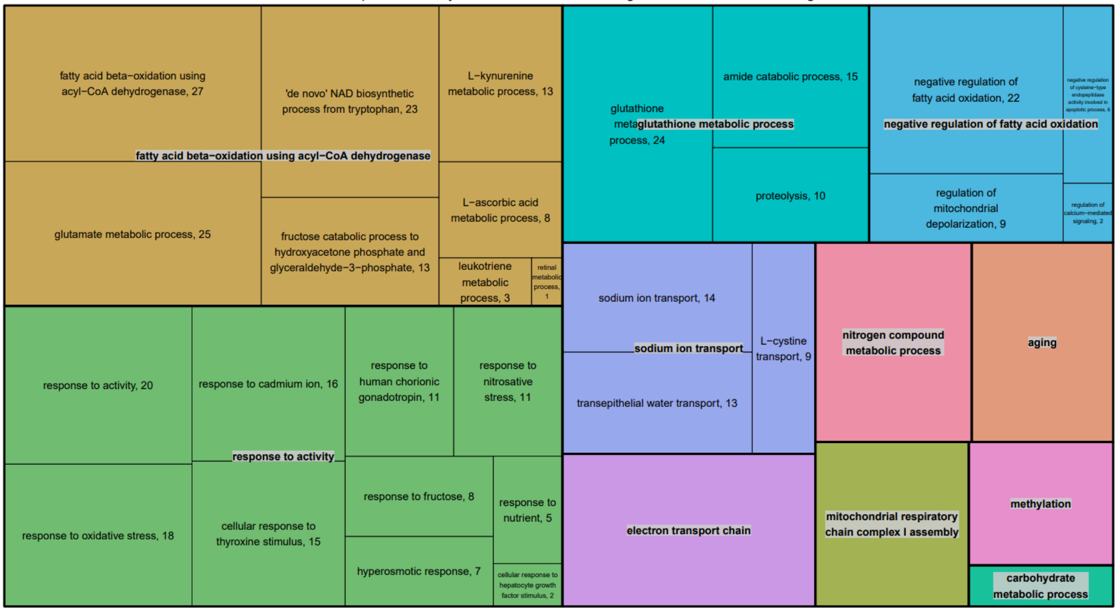

Proteomics and Transcriptomics Kidney Studies 1&2 Down-Regulated Intersection GO Molecular Function

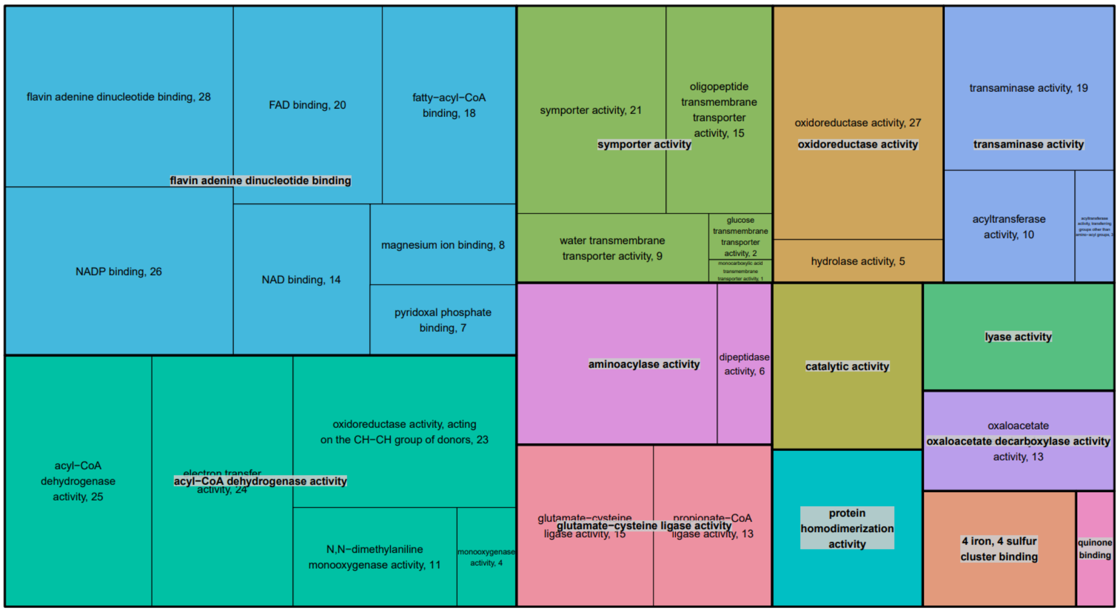

Proteomics and Transcriptomics Kidney Studies 1&2 Down-Regulated Intersection GO Cellular Component

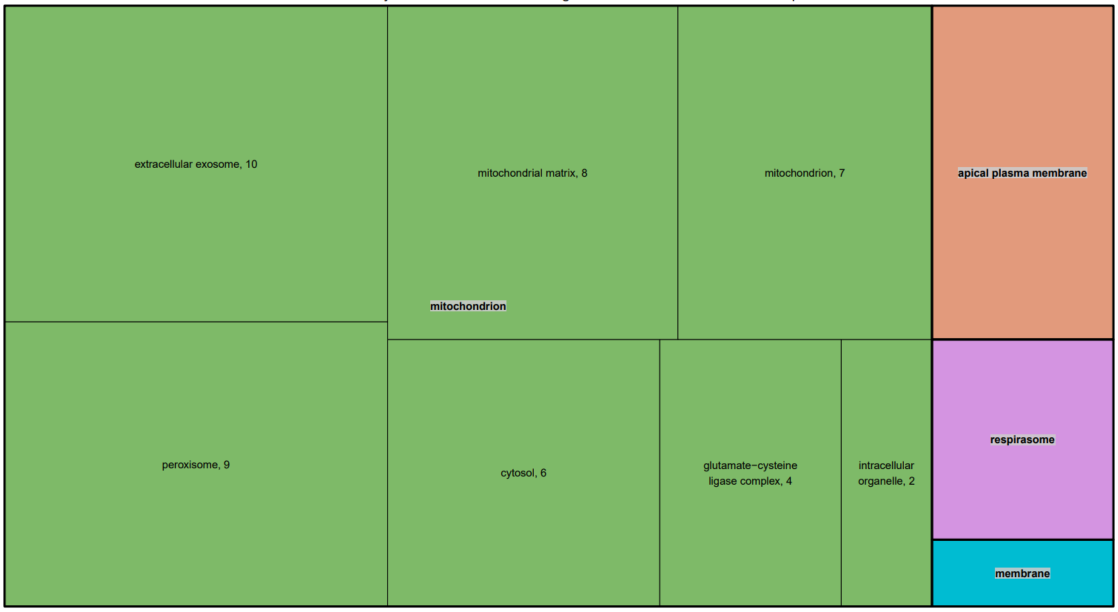

Supplementary Figure 7: Protein and gene expression of proteins in common in all proteomic and transcriptomic datasets. **a)** Additional up-regulated proteins (plasma proteins excluded). **b)** Down-regulated proteins.

a

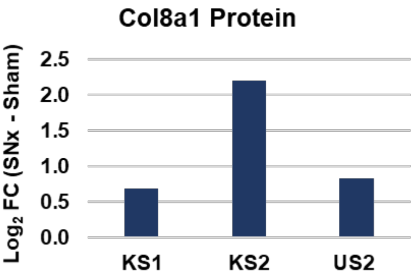

Tissue	log_2FC	P-Value	FDR
KS1	0.68	6.97E-03	4.36E-02
KS2	2.19	7.25E-04	2.85E-03
US2	0.84	3.67E-03	6.68E-03

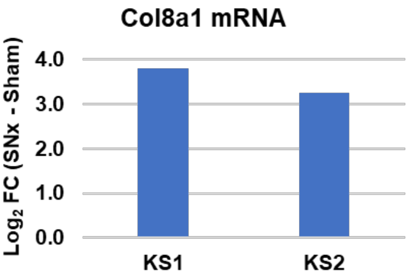

	log_2FC	P-Value	FDR
KS1	3.79	4.62E-05	9.20E-04
KS2	3.25	2.15E-07	2.76E-06

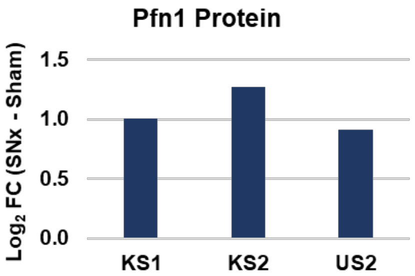

Tissue	log_2FC	P-Value	FDR
KS1	1.01	1.13E-03	1.94E-02
KS2	1.27	2.59E-05	4.08E-04
US2	0.91	3.03E-02	4.36E-02

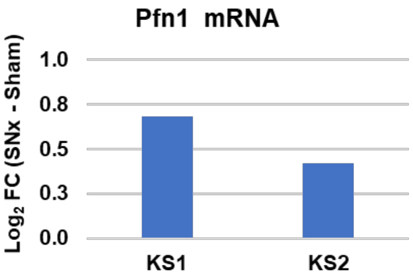

Tissue	log_2FC	P-Value	FDR
KS1	0.68	6.38E-04	3.94E-03
KS2	0.42	4.76E-04	1.01E-03

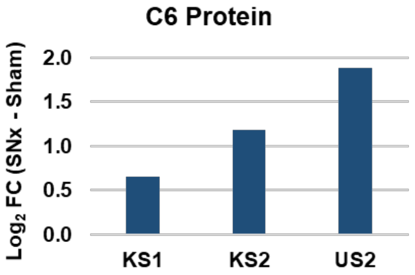

Tissue	log_2FC	P-Value	FDR
KS1	0.66	4.39E-03	4.04E-02
KS2	1.18	4.77E-05	2.88E-02
US2	1.88	2.62E-05	3.17E-03

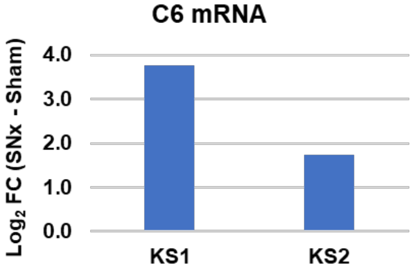

Tissue	log_2FC	P-Value	FDR
KS1	3.76	6.15E-03	1.94E-02
KS2	1.73	5.21E-03	8.59E-03

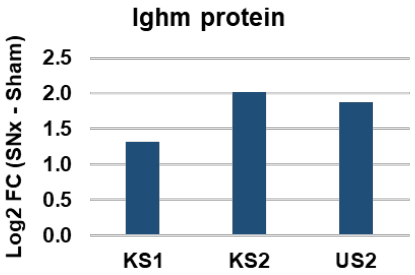

Tissue	log_2FC	P-Value	FDR
KS1	1.31	3.28E-04	1.17E-02
KS2	2.03	9.46E-04	3.40E-03
US2	1.88	2.62E-05	3.17E-03

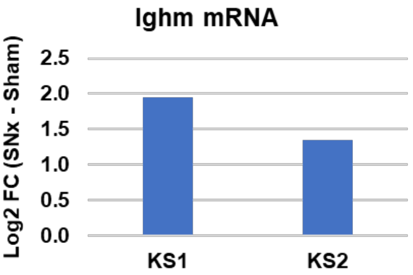

Tissue	log_2FC	P-Value	FDR
KS1	1.95	1.68E-03	7.56E-03
KS2	1.35	2.62E-02	3.71E-02

b

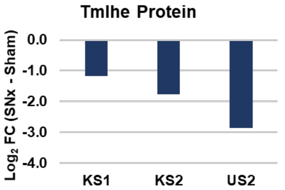

Tissue	log2FC	P-Value	FDR
KS1	-1.17	5.78E-03	4.04E-02
KS2	-1.77	1.57E-02	2.88E-02
US2	-2.86	1.50E-03	3.17E-03

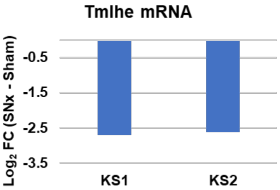

Tissue	log2FC	P-Value	adj.P-Val
KS1	-2.71	2.44E-05	7.32E-04
KS2	-2.62	5.55E-08	1.31E-06

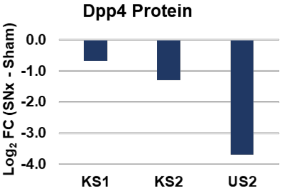

Tissue	log2FC	P-Value	FDR
KS1	-0.70	4.27E-03	3.54E-02
KS2	-1.31	1.96E-02	3.43E-02
US2	-3.70	2.26E-07	1.93E-06

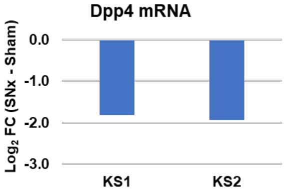

Tissue	log2FC	P-Value	adj.P-Val
KS1	-1.81	1.80E-05	6.67E-04
KS2	-1.92	6.07E-09	4.81E-07

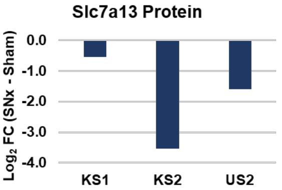

Tissue	log2FC	P-Value	FDR
KS1	-0.56	2.18E-03	2.67E-02
KS2	-3.54	1.83E-06	1.37E-04
US2	-1.60	1.30E-03	2.80E-03

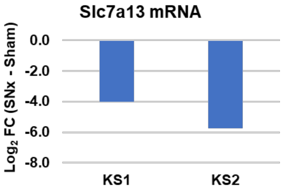

Tissue	log2FC	P-Value	adj.P-Val
KS1	-4.01	1.56E-03	7.19E-03
KS2	-5.75	5.35E-06	2.45E-05

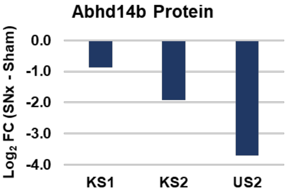

Tissue	log2FC	P-Value	FDR
KS1	-0.86	2.60E-03	2.85E-02
KS2	-1.93	1.47E-02	2.72E-02
US2	-3.69	3.97E-04	1.04E-03

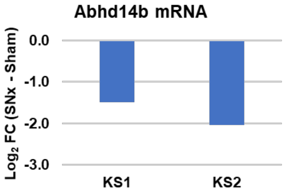

Tissue	log2FC	P-Value	adj.P-Val
KS1	-1.49	1.21E-03	5.99E-03
KS2	-2.05	6.50E-07	5.54E-06

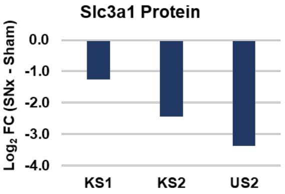

Tissue	log2FC	P-Value	FDR
KS1	-1.25	1.99E-03	2.55E-02
KS2	-2.44	9.81E-03	1.99E-02
US2	-3.36	1.64E-05	7.19E-05

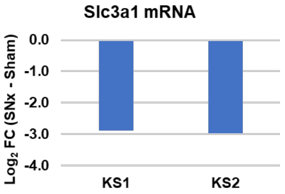

Tissue	log2FC	P-Value	adj.P-Val
KS1	-2.88	2.12E-04	1.99E-03
KS2	-2.98	4.84E-07	4.67E-06

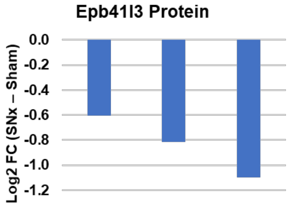

Tissue	log2FC	P-Value	FDR
KS1	-0.61	6.61E-03	4.28E-02
KS2	-0.81	7.79E-05	7.11E-04
US2	-1.10	9.23E-03	1.52E-02

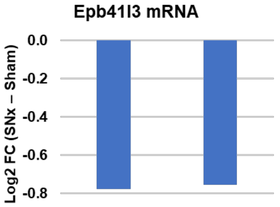

Tissue	log2FC	P-Value	FDR
KS1	-0.78	9.77E-04	5.20E-03
KS2	-0.76	1.01E-02	1.56E-02

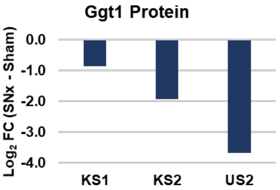

Tissue	log2FC	P-Value	FDR
KS1	-0.86	2.60E-03	2.85E-02
KS2	-1.93	1.47E-02	2.72E-02
US2	-3.69	3.97E-04	1.04E-03

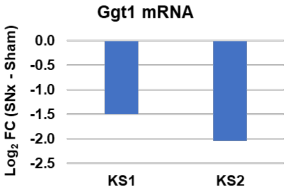

Tissue	log2FC	P-Value	adj.P-Val
KS1	-1.49	1.21E-03	5.99E-03
KS2	-2.05	6.50E-07	5.54E-06

Supplementary Figure 8: Next Bio Human Kidney Disease gene expression database repository. Lumican and Col3a1 gene expression in kidneys compared to controls in diseased patients.

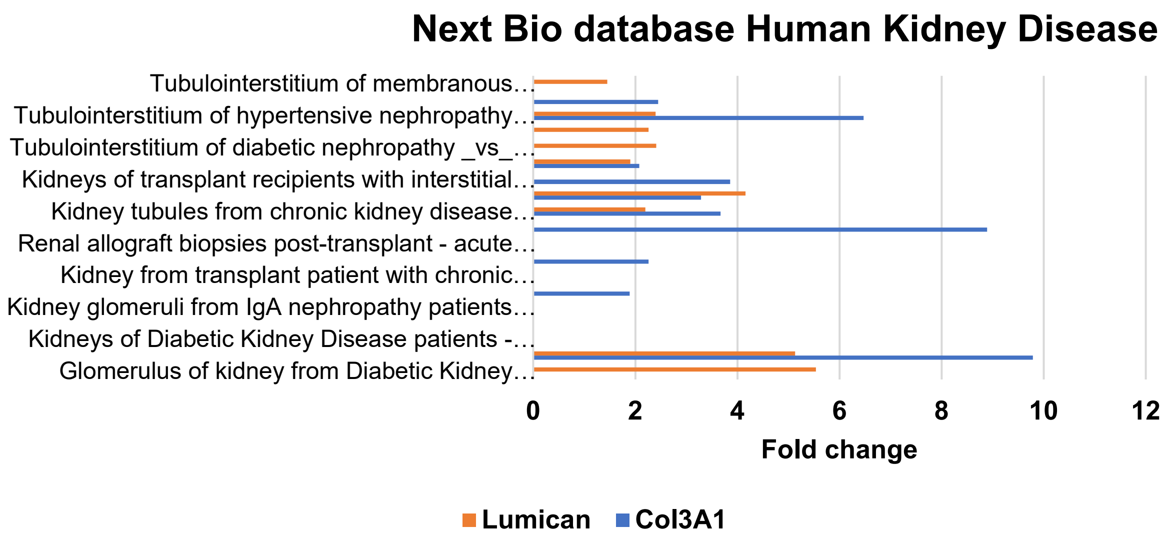

Supplement: Supplementary file 1 — Supplementary Information 1. [file 41598_2025_93894_MOESM1_ESM.pdf]
